# Efficacy and safety of nelfinavir in asymptomatic and mild COVID-19 patients: a structured summary of a study protocol for a multicenter, randomized controlled trial

**DOI:** 10.1186/s13063-021-05282-w

**Published:** 2021-04-28

**Authors:** Naoki Hosogaya, Taiga Miyazaki, Yuri Fukushige, Sachiko Takemori, Shinpei Morimoto, Hiroshi Yamamoto, Makoto Hori, Tomoya Kurokawa, Yohei Kawasaki, Michiko Hanawa, Yasuhisa Fujii, Hideki Hanaoka, Shingo Iwami, Koichi Watashi, Satoshi Yamagoe, Yoshitsugu Miyazaki, Takaji Wakita, Koichi Izumikawa, Katsunori Yanagihara, Hiroshi Mukae, Shigeru Kohno, Nelfinavir Study Group, Hiroshi Yotsuyanagi, Yasuyuki Kato, Tetsuya Matsumoto, Norihito Tarumoto, Satoshi Shiraishi, Hiroshi Ishii, Hideaki Ohno, Kazuhiro Yatera, Yoshihiro Yamamoto, Hiroshi Kakeya

**Affiliations:** 1 Clinical Research Center, Nagasaki University Hospital, Nagasaki, Japan; 2 Department of Respiratory Medicine, Nagasaki University Hospital, Nagasaki, Japan; 3 Department of Infectious Diseases, Nagasaki University Graduate School of Biomedical Sciences, 1-7-1 Sakamoto, Nagasaki, 852-8501 Japan; 4 Department of Hospital Pharmacy Nagasaki University Hospital, Nagasaki, Japan; 5 Clinical Research Center, Chiba University Hospital, Chiba, Japan; 6 Department of Biology, Faculty of Sciences, Kyushu University, Fukuoka, Japan; 7 Department of Virology II, National Institute of Infectious Diseases, Tokyo, Japan; 8 Research Center for Drug and Vaccine Development, National Institute of Infectious Diseases, Tokyo, Japan; 9 Department of Chemotherapy and Mycoses, National Institute of Infectious Diseases, Tokyo, Japan; 10 Department of Laboratory Medicine, Nagasaki University Hospital, Nagasaki, Japan; 11 Department of Respiratory Medicine, Nagasaki University Graduate School of Biomedical Sciences, Nagasaki, Japan

**Keywords:** nelfinavir, COVID-19, SARS-CoV-2, asymptomatic, mild, blinded outcome assessment, randomized controlled trial, protocol

## Abstract

**Objectives:**

The aim of this trial is to evaluate the antiviral efficacy, clinical efficacy, and safety of nelfinavir in patients with asymptomatic and mild COVID-19.

**Trial design:**

The study is designed as a multicenter, open-label, blinded outcome assessment, parallel group, investigator-initiated, exploratory, randomized (1:1 ratio) controlled clinical trial.

**Participants:**

Asymptomatic and mild COVID-19 patients will be enrolled in 10 university and teaching hospitals in Japan. The inclusion and exclusion criteria are as follows:

Inclusion criteria:

1. Japanese male or female patients aged ≥ 20 years
2. SARS-CoV-2 detected from a respiratory tract specimen (e.g., nasopharyngeal swab or saliva) using PCR, LAMP, or an antigen test within 3 days before obtaining the informed consent
3. Provide informed consent

Exclusion criteria:

1. Symptoms developed ≥ 8 days prior to enrolment
2. SpO_2_ < 96 % (room air)
3. Any of the following screening criteria:

- ALT or AST ≥ 5 × upper limit of the reference range
- Child-Pugh class B or C
- Serum creatinine ≥ 2 × upper limit of the reference range and creatinine clearance < 30 mL/min

(4) Poorly controlled diabetes (random blood glucose ≥ 200 mg/dL or HbA1c ≥ 7.0%, despite treatment)
(5) Unsuitable serious complications based on the assessment of either the principal investigator or the sub-investigator
(6) Hemophiliac or patients with a marked hemorrhagic tendency
(7) Severe diarrhea
(8) Hypersensitivity to the investigational drug
(9) Breastfeeding or pregnancy
(10) With childbearing potential and rejecting contraceptive methods during the study period from the initial administration of the investigational drug
(11) Receiving rifampicin within the previous 2 weeks
(12) Participated in other clinical trials and received drugs within the previous 12 weeks
(13) Undergoing treatment for HIV infection
(14) History of SARS-CoV-2 vaccination or wishes to be vaccinated against SARS-CoV-2
(15) Deemed inappropriate (for miscellaneous reasons) based on the assessment of either the principal investigator or the sub-investigator

**Intervention and comparator:**

Patients who meet the inclusion criteria and do not meet any of the exclusion criteria will be randomized to either the nelfinavir group or the symptomatic treatment group.

The nelfinavir group will be administered 750 mg of nelfinavir orally, three times daily for 14 days (treatment period). However, if a participant tests negative on two consecutive PCR tests of saliva samples, administration of the investigational drug for that participant can be discontinued at the discretion of the investigators.

The symptomatic treatment group will not be administered the investigational drug, but all other study procedures and conditions will be the same for both groups for the duration of the treatment period. After the treatment period of 14 days, each group will be followed up for 14 days (observational period).

**Main outcomes:**

The primary endpoint is the time to negative conversion of SARS-CoV-2. During the study period from Day 1 to Day 28, two consecutive negative PCR results of saliva samples will be considered as the negative conversion of the virus.

The secondary efficacy endpoints are as follows:

For patients with both asymptomatic and mild disease: area under the curve of viral load, half decay period of viral load, body temperature at each time point, all-cause mortality, incidence rate of pneumonia, percentage of patients with newly developed pneumonia, rate of oxygen administration, and the percentage of patients who require oxygen administration.

For asymptomatic patients: incidence of symptomatic COVID-19, incidence of fever (≥ 37.0 °C for two consecutive days), incidence of cough

For patients with mild disease: incidence of defervescence (< 37.0 °C), incidence of recovery from clinical symptoms, incidence of improvement of each symptom

The secondary safety endpoints are adverse events and clinical examinations.

**Randomization:**

Patients will be randomized to either the nelfinavir group or the symptomatic treatment group using the electric data capture system (1:1 ratio, dynamic allocation based on severity [asymptomatic], and age [< 60 years]).

**Blinding (masking):**

Only the assessors of the primary outcome will be blinded (blinded outcome assessment).

**Numbers to be randomized (sample size):**

The sample size was determined based on our power analysis to reject the null hypothesis, S (t | z =1) = S (t | z = 0) where S is a survival function, t is time to negative conversion, and z denotes randomization group, by the log-rank test with a two-sided p value of 0.05. We estimated viral dynamic parameters by fitting a nonlinear mixed-effects model to reported viral load data, and simulated our primary endpoint from viral-load time-courses that were realized from sets of viral dynamics parameters sampled from the estimated probability distribution of the parameters (sample size: 2000; 1000 each for randomization group). From this estimation of the hazard ratio between the randomization groups for the event of negative conversion using this simulation dataset, the required number of events for rejecting our null hypothesis with a power of 0.80 felled 97.345 by plugging the estimated hazard ratio, 1.79, in Freedman’s equation. Therefore, we decided the required number of randomizations to be 120 after consideration of the frequency of censoring and the anticipated rate of withdrawal caused by factors such as withdrawal of consent.

**Trial Status:**

Protocol version 6.0 of February 12, 2021. Recruitment started on July 22, 2020 and is anticipated to be completed by March 31, 2022.

**Trial registration:**

This trial was registered in Japan Registry of Clinical Trials (jRCT) (jRCT2071200023) on 21 July 21, 2020.

**Full protocol:**

The full protocol is attached as an additional file, accessible from the Trials website (Additional file 1). In the interest in expediting dissemination of this material, the familiar formatting has been eliminated; this Letter serves as a summary of the key elements of the full protocol.

The study protocol has been reported in accordance with the Standard Protocol Items: Recommendations for Clinical Interventional Trials (SPIRIT) guidelines (Additional file 2).

**Supplementary Information:**

The online version contains supplementary material available at 10.1186/s13063-021-05282-w.

## Supplementary Information

**Additional file 1.** Full study protocol.

**Additional file 2.** SPIRIT 2013 Checklist: Recommended items to address in a clinical trial protocol and related documents*.

## Data Availability

The full study protocol is available in the supplementary materials and at https://jrct.niph.go.jp/en-latest-detail/jRCT2071200023. The data are not available because the trial is in progress. The data will be made available from the author on reasonable request once the trial has been completed. Please contact the corresponding author, Dr. T. Miyazaki (taiga-m@nagasaki-u.ac.jp).

