# Supplementary material for: Efficacy and safety of nelfinavir in asymptomatic and mild COVID-19 patients: a structured summary of a study protocol for a multicenter, randomized controlled trial
Source: Trials. 2021 Apr 28;22:309. doi: 10.1186/s13063-021-05282-w (PMC8080096; doi:10.1186/s13063-021-05282-w)
Supplement: Supplementary file 1 — Additional file 1. Full study protocol. [file 13063_2021_5282_MOESM1_ESM.pdf]

無症状及び軽症 COVID-19 患者に対する
ネルフィナビルの有効性及び安全性を探索する
ランダム化非盲検並行群間比較試験

治験実施計画書

治験調整医師

長崎大学大学院医歯薬学総合研究科
臨床感染症学分野

宮崎　泰可

秘密保持条項

本治験実施計画書に含まれる情報は機密情報であり、本治験実施計画書は本治験に参加いただく治験責任医師、各実施医療機関の関係者、治験薬提供者、及び治験審査委員会に限り提供するものです。したがって、被験者から同意を得る場合、又は治験分担医師、治験協力者に説明する場合を除き、自ら治験を実施する者の許可なしには、本治験実施計画書に含まれる情報を第三者に開示することはできません。

承　認　者	署　　　名	日　　　付
治験調整医師	宮崎 泰可	2021, 2, 12
治験調整事務局責任者	福重 友理	2021、2、12

改訂履歴

版番号	改訂日	作成者	改訂理由／内容
6.0	2021年2月12日	宮崎　泰可	除外基準改訂、併用禁止薬改訂、記載整備
5.1	2020年12月24日	宮崎　泰可	治験期間延長
5.0	2020年11月11日	宮崎　泰可	選択基準改訂、収集項目追加
4.1	2020年10月5日	宮崎　泰可	医学的に重要な事象について追記<br>誤記修正、記載整備
4.0	2020年8月19日	宮崎　泰可	適切な評価方法を再考の上、修正追記<br>治験薬提供者追記<br>誤記修正、記載整備
3.1	2020年7月20日	宮崎　泰可	適切な評価方法を再考の上、修正追記
3.0	2020年7月8日	宮崎　泰可	適切な評価方法を再考の上、修正追記<br>原資料の保存に関する取扱いについて明記、<br>誤記修正、記載整備
2.0	2020年6月12日	宮崎　泰可	適切な評価方法を再考の上、修正追記<br>誤記修正、記載整備
1.0	2020年5月18日	宮崎　泰可	新規作成

略語

略語	英名	和名
AE	Adverse Event	有害事象
ALP	Alkaline phosphatase	アルカリフォスファターゼ
ALT	Alanine aminotransferase	アラニンアミノトランスフェラーゼ
AST	Aspartate aminotransferase	アスパラギン酸アミノトランスフェラーゼ
BUN	Blood urea nitrogen	血中尿素窒素
CK	Creatine Kinase	クレアチンキナーゼ
CONSORT	Consolidated standards of reporting trials	臨床試験報告に関する統合基準
COVID-19	Coronavirus Disease 2019	新型コロナウイルス感染症
CRE	Creatinine	クレアチニン
CRP	C-reactive protein	C 反応性蛋白
EDC	Electronic data capture	電子的データ収集システム
FAS	Full analysis set	最大解析対象集団
GCP	Good clinical practice	医薬品の臨床試験の実施に関する基準
HIV	Human Immunodeficiency Virus	ヒト免疫不全ウイルス
NEWS2	National Early Warning Score 2	早期警告スコア 2
PCR	Polymerase chain reaction	ポリメラーゼ連鎖反応
PPS	Per protocol set	治験実施計画書に適合した対象集団
PT	Preferred terms	基本語
RNA	ribonucleic acid	リボ核酸
SARS-CoV-2	Severe Acute Respiratory Syndrome Coronavirus 2	重症急性呼吸器症候群コロナウイルス 2
SAS	Safety analysis set	安全性解析対象集団
SOC	System organ class	器官別大分類
SpO_2	oxyhemoglobin saturation measured by pulse oximetry	経皮的動脈血酸素飽和度
T-bil	Total bilirubin	総ビリルビン
γ-GTP	γ-Glutamyltranspeptidase	γ グルタミン酸トランスペプチダーゼ

目次

1 背景情報 - 14 -
1.1 はじめに - 14 -
1.1.1 ネルフィナビルの概略 - 14 -
1.1.2 新型コロナウイルス感染症（COVID-19）の病態 - 14 -
1.1.3 COVID-19 の診断、治療 - 14 -
1.2 治験に関する背景情報 - 14 -
1.2.1 背景 - 14 -
1.2.2 被験薬選択の根拠 - 15 -
1.2.3 本治験計画の根拠 - 15 -
1.2.4 海外での臨床試験及び開発状況 - 15 -
1.2.5 国内での臨床試験及び開発状況 - 15 -
1.2.6 本治験の妥当性 - 15 -
1.2.7 医薬品の臨床試験の実施の基準に関する省令（GCP）遵守に関する陳述 - 16 -
2 治験の目的 - 16 -
3 治験方法 - 16 -
3.1 治験のデザイン - 16 -
3.1.1 治験デザイン設定の根拠 - 16 -
3.2 対象疾患 - 16 -
3.2.1 対象集団選択の根拠 - 16 -
3.3 目標被験者数・治験薬投与期間 - 17 -
3.3.1 目標被験者数・治験薬投与期間 - 17 -
3.3.2 目標被験者数の設定根拠 - 17 -
3.4 評価項目 - 17 -
3.4.1 主要評価項目 - 17 -
3.4.1.1 主要評価項目の設定根拠 - 17 -
3.4.2 副次評価項目 - 17 -
3.4.2.1 副次評価項目の設定根拠 - 18 -
3.4.3 探索的項目 - 18 -
3.5 治験の手順 - 19 -
3.5.1 被験者の選定からスクリーニング期までの手順 - 19 -
3.5.2 被験者の適格性判定及び登録の手順 - 19 -
3.5.3 登録以降の手順 - 19 -
3.6 各被験者の治験期間 - 20 -
3.7 治験実施予定期間 - 20 -
4 被験者の選択、除外、中止基準 - 20 -
4.1 選択基準 - 20 -
4.2 除外基準 - 20 -
4.3 中止基準 - 21 -
4.3.1 被験者の治験の打ち切り - 21 -
4.3.2 中止時の対処 - 21 -
5 治験薬 - 21 -
5.1 被験薬及び対照薬 - 21 -
5.2 包装及び表示 - 21 -
5.3 治験薬の保管・管理手順 - 22 -
5.4 その他、治験薬以外の実施医療機関への提供物 - 22 -
6 被験者に対する治療 - 22 -
6.1 治験薬の投与 - 22 -

6.1.1 期間及び用法・用量 - 22 -
6.1.1.1 治験期間の設定根拠 - 22 -
6.1.2 投与方法 - 22 -
6.1.2.1 被験薬の用法・用量の設定根拠 - 22 -
6.1.3 被験薬の休薬基準 - 23 -
6.1.4 被験薬の中止基準 - 23 -
6.2 観察・検査スケジュール - 24 -
6.2.1 治験スケジュール - 24 -
6.2.2 観察・検査項目 - 25 -
6.3 試料の採取及び測定 - 27 -
6.3.1 唾液検体の取扱い - 27 -
6.4 前治療及び併用薬・併用療法等 - 27 -
6.4.1 下痢の対処法 - 27 -
6.4.2 併用が禁止される薬剤 - 28 -
6.5 過量投与 - 28 -
6.6 被験者の管理 - 28 -
6.7 治療方法遵守の確認 - 28 -
7 有害事象 - 29 -
7.1 有害事象発生時の対応 - 29 -
7.1.1 有害事象の定義 - 29 -
7.1.2 有害事象の報告 - 29 -
7.1.3 重篤な有害事象 - 29 -
7.1.4 有害事象の治験薬との因果関係 - 29 -
7.1.5 有害事象の転帰 - 30 -
7.1.6 副作用 - 30 -
7.1.7 有害事象の重症度の判定 - 30 -
7.1.8 有害事象の重篤性の判定 - 30 -
7.1.9 有害事象の治療のためにとられた処置 - 30 -
7.2 有害事象の記録と報告 - 30 -
7.2.1 有害事象の記録 - 30 -
7.2.2 重篤な有害事象の報告 - 31 -
7.2.3 妊娠に関する報告 - 31 -
7.2.4 緊急報告 - 31 -
7.3 有害事象発現時の被験者のフォローアップ - 31 -
7.4 効果安全性評価委員会 - 31 -
7.5 医学的に重要な事象 - 31 -
7.5.1 薬物誘発性肝障害の可能性(Hy’s Law) - 31 -
7.5.2 授乳期間中の治験薬曝露 - 32 -
7.5.3 職業的治験薬曝露 - 32 -
7.5.4 投薬過誤 - 32 -
8 統計解析 - 33 -
8.1 全般的事項 - 33 -
8.2 解析対象集団の定義 - 33 -
8.3 症例の取り扱い - 33 -
8.4 被験者特性の解析 - 33 -
8.5 有効性の解析 - 34 -
8.5.1 主要評価項目 - 34 -
8.5.2 副次評価項目 - 34 -
8.6 安全性の解析 - 34 -
9 原資料の特定/原資料等の直接閲覧 - 35 -

9.1 原資料の特定 - 35 -
9.2 外部測定機関から直接入手するデータ - 35 -
9.3 画像評価の回収 - 35 -
9.4 原資料等の直接閲覧 - 35 -
10 治験の品質管理及び品質保証 - 35 -
10.1 治験の品質管理及び品質保証 - 35 -
10.2 本治験における品質管理及び品質保証 - 35 -
10.3 治験調整医師の監査と規制当局の査察 - 36 -
10.4 利益相反について - 36 -
11 倫理 - 36 -
11.1 被験者への説明と同意の取得 - 36 -
11.1.1 説明文書の作成 - 36 -
11.1.2 説明文書に記載すべき内容 - 36 -
11.1.3 同意取得の時期と方法 - 37 -
11.1.4 説明文書及び同意文書の改訂 - 37 -
11.2 治験審査委員会 - 37 -
11.3 被験者の人権保護 - 37 -
11.4 同意撤回時の対応 - 38 -
11.5 治験終了後の対応 - 38 -
11.6 被験者に対する開示 - 38 -
12 データ及び試料の取扱いと保存 - 38 -
12.1 症例報告書の作成、変更・修正 - 38 -
12.2 試料・情報の保管管理 - 38 -
12.2.1 試料・情報の取扱い - 38 -
12.2.2 保管期間・場所 - 38 -
12.2.3 保管期間終了後の廃棄方法 - 39 -
12.3 試料・情報の二次利用 - 39 -
12.4 記録の保存 - 39 -
12.4.1 治験審査委員会 - 39 -
12.4.2 実施医療機関 - 39 -
12.4.3 治験責任医師又は治験分担医師 - 39 -
12.4.4 治験調整医師 - 39 -
13 金銭の支払い及び健康被害補償 - 40 -
13.1 治験の費用 - 40 -
13.2 健康被害補償 - 40 -
14 成果の帰属と公表に関する取り決め - 40 -
15 治験実施計画書の変更 - 40 -
15.1 治験実施計画書の改訂 - 40 -
15.2 治験実施計画書からの逸脱・変更 - 40 -
16 治験の中止・中断・終了 - 41 -
16.1 治験の中止・中断 - 41 -
16.1.1 治験全体の中止・中断 - 41 -
16.1.2 各実施医療機関における治験の中止・中断 - 41 -
16.2 治験の終了 - 41 -
17 治験実施体制 - 41 -
18 参考資料及び文献 - 41 -

付録 1. 服薬日誌　 (2020 年 6 月 12 日作成)

付録 2. 症状日誌　 (2020 年 7 月 8 日作成)

0. 概要

治験の標題	
治験課題名	無症状及び軽症 COVID-19 患者に対するネルフィナビルの有効性及び安全性を探索するランダム化非盲検並行群間比較試験
治験実施計画書番号	NUH05COVID-19
治験薬名	AG-1343 錠 250 mg
治験実施計画書の要約	
治験の目的	無症状及び軽症 COVID-19 患者において、被験薬投与群及び対症療法群における重症急性呼吸器症候群コロナウイルス 2（SARS-CoV-2）ウイルス量の変化、臨床症状の改善、有害事象発現を評価し、AG-1343 の COVID-19 に対する抗ウイルス効果、臨床的有効性及び安全性を評価する。
対象疾患	無症状及び軽症 COVID-19
目標症例数	120 例（被験薬投与群及び対症療法群各 60 例）
選択基準	無症状及び軽症 COVID-19 患者で、以下のすべての基準を満たす場合に登録可とする。<br>(1) 同意取得時に 20 歳以上の日本人患者、男女の性別は問わない<br>(2) 同意取得前 3 日以内に上下気道由来検体から PCR 検査又は LAMP 法で SARS-CoV-2 が検出された患者、又は抗原検査が陽性であった患者<br>(3) 説明文書の内容、その他、治験に関する事項について十分な説明を受け、その内容を理解し、本治験への参加について自由意思に基づく文書による同意が得られた患者
除外基準	以下のうち一つでも該当する患者は対象から除外する。<br>(1) 登録時点で症状出現から 8 日以上経過している患者<br>(2) 経皮酸素飽和度（SpO_2）が 96%未満（室内気）の患者<br>(3) スクリーニング時検査において以下のいずれかに該当する患者<br>1） ALT 又は AST が基準値上限の 5 倍を超える<br>2） Child-Pugh 分類 B 又は C に該当する<br>3） 血清クレアチニンが基準値上限の 2 倍を超え、且つクレアチニンクリアランスが 30 mL/min 未満*の場合<br>*Cockcroft-Gault 式による推定クレアチニンクリアランス値。ただし実測値がある場合は実測値を用いる。<br>(4) コントロール不良の糖尿病患者（糖尿病の治療を受けているにもかかわらず、随時血糖が 200 mg/dL 以上又は HbA1c が 7.0 %以上の糖尿病患者）<br>(5) 重篤な疾患を合併しており、治験対象として治験責任医師又は治験分担医師が不適当と判断した患者<br>(6) 血友病患者又は著しい出血傾向のある患者<br>(7) 高度な下痢症状がある患者<br>(8) 被験薬の成分に対し過敏症の既往歴のある患者<br>(9) 授乳中、妊娠中、又は妊娠の可能性がある女性患者<br>(10) 被験薬初回投与日から治験参加の期間、適切な避妊法を用いることができない患者（男性及び女性は妊娠の可能性がある女性）<br>(11) リファンピシンを同意取得前 2 週間以内に服用した患者<br>(12) 同意取得前 12 週以内に他の治験又は臨床試験に参加し投薬を受けた患者<br>(13) HIV 感染症治療中の患者<br>(14) 新型コロナワクチンの接種歴のある患者、治験参加中に新型コロナワクチンの接種を希望する患者<br>(15) その他、治験責任医師あるいは治験分担医師が不適当と判断した患者
治験デザイン	医師主導、多施設共同ランダム化非盲検並行群間比較試験
治験の相	I/II 相

治験参加予定期間	最大 28 日間（14 日間又は 15 日間の治験薬投与期間、14 日間の追跡期間）
投与開始基準	スクリーニング時および登録時/Day1 の検査・観察・評価において選択基準に合致し除外基準に抵触しないことが確認できた患者に対し、ランダム割付を行い、投与を開始する。
治験薬の用量及び投与方法	1 回 750 mg を 1 日 3 回経口投与する。ただし、休薬は基準に従う。（ただし、2 回連続で唾液 PCR 陰性が確認された場合、治験責任医師又は治験分担医師の判断で、治験薬服用は終了可能とする）
被験薬の休薬基準	下記の基準に従って被験薬の休薬を行う。<br>● 被験薬と因果関係があると治験責任医師又は治験分担医師が判断し、高度又は医学的に重大、若しくは許容できないと判断した有害事象が認められた場合<br>● その他安全性上問題があると治験責任医師又は治験分担医師が判断した場合
被験薬の中止基準	下記の基準に従って被験薬の投与を中止する。<br>● 治験責任医師又は治験分担医師が治験薬と因果関係があると判断した、高度又は医学的に重大である有害事象が、治験薬の休薬後 4 日以内に中等度以下まで回復しない場合<br>● その他、安全性上問題があると治験責任医師又は治験分担医師が判断した場合
治験中止基準	(1) 被験者が同意を撤回した場合<br>(2) 生命を脅かす：緊急処置を要する、有害事象が発現した場合<br>(3) COVID-19 の悪化により、治験責任医師又は治験分担医師が治験の継続が困難又は適切でないと判断された場合<br>(4) その他治験責任医師、治験分担医師、又は効果安全性評価委員会により治験の継続が困難又は適切でないと判断された場合
評価項目	● 主要評価項目<br>登録時からウイルス陰性化までの日数<br><br>● 副次評価項目<br>有効性副次評価項目は、無症状者、軽症者それぞれに設定する。<br><br>● 有効性評価項目<br>＜共通項目＞<br>(1) ウイルス量の曲線下面積：ウイルス量の曲線下面積は、登録時から 28 日間における唾液 PCR 検査によるウイルス量より算出する。<br>(2) ウイルス量の半減期：ウイルス量の半減期は、登録時から 28 日間における唾液 PCR 検査によるウイルス量より算出する。<br>(3) 各時点における体温<br>(4) 全死亡者の割合：全死亡者の割合は、登録時から 28 日間での全死因による死亡の有無より算出する。<br>(5) 肺炎発症率：肺炎発症率は、登録時から肺炎発症までの日数より算出する。<br>(6) 肺炎発症者の割合：肺炎発症者の割合は、登録時から 28 日後までの COVID-19 によると判断される肺炎の発症の有無より算出する。<br>(7) 酸素投与率：酸素投与率は、登録時から「24 時間以上持続する酸素投与」の開始までの日数より算出する。<br>(8) 酸素投与者の割合：酸素投与者の割合は、登録時から 28 日後までの「24 時間以上持続する酸素投与」の開始の有無より算出する。<br><br>＜無症状者＞<br>(1) COVID-19 発症者の割合：COVID-19 発症者の割合は、登録時から 28 日間での COVID-19 によると判断される「2 日連続する 37.0°C以上の発

	熱」、「24 時間以上持続する咳嗽」を含め何れかの症状の発現の有無より算出する。<br>(2) 発熱症状を発現した被験者の割合：発熱症状を発現した被験者の割合は、登録時から 28 日間での「2 日連続する 37.0℃以上の発熱」の発現の有無より算出する。<br>(3) 咳嗽症状を発現した被験者の割合：咳嗽症状を発現した被験者の割合は、登録時から 28 日間での「24 時間以上持続する咳嗽」の発現の有無より算出する。<br><br>＜軽症者＞<br>(1) 解熱率：解熱率は、登録時から体温 37.0℃未満に回復するまでの日数より算出する。<br>(2) 臨床症状の全消失率：臨床症状の全消失率は、COVID-19 によると判断される全ての臨床症状が消失するまでの日数より算出する。<br>(3) 症状ごとの改善率：症状ごとの改善率は、COVID-19 によると判断される臨床症状が改善するまでの日数（症状の内容ごとに評価）より算出する。<br><br>肺炎の定義：胸部単純 X 線又は胸部 CT において肺炎像が出現した場合<br>COVID-19 発症の定義：唾液 PCR 検査が陽性であり、且つ COVID-19 によると判断される何らかの臨床症状が発現した場合<br>「24 時間以上持続する咳嗽」の定義：症状日誌（付録 2）にて咳嗽について「あり」が連続して 2 回以上続いているとき、そのうち 1 回目の記録を「24 時間以上持続する咳嗽」:「あり」とする。<br>症状改善の定義：体温 37.0℃未満、SpO_2≧96%（室内気）、その他の症状は症状が 5 割以上軽減した状態が 48 時間以上持続した場合<br><br>● 安全性評価項目<br>(1) 有害事象（有害事象発現割合、重篤な有害事象発現割合、副作用発現割合、重篤な副作用発現割合、）<br>(2) 臨床検査（血液学的検査、血液生化学検査、バイタルサイン）<br><br>● 探索的項目<br>NEWS2
下痢の対処法	被験薬に特化した対処法は存在しないので、ロペラミドやタンニン酸製剤等の投与を行う。ただし、COVID-19 の症状としても下痢が認められることがあるため、慎重に判断すること。
併用禁止薬	<<共通>><br>他の臨床試験薬の投与、臨床試験機器の使用<br>新型コロナワクチン<br><br><<被験薬投与群のみ>><br>以下の薬剤は併用禁止：トリアゾラム、ミダゾラム、アルプラゾラム、ピモジド、バッカク誘導体、アミオダロン、キニジン硫酸塩水和物、ブロナンセリン、スボレキサント、イバブラジン、チカグレロル、リバーロキサバン、アゼルニジピン、リファンピシン、エレトリプタン臭化水素酸塩、エプレレノン、インジナビル硫酸塩エタノール付加物、サキナビル酸塩、リトナビル、ホスアンプレナビルカルシウム水和物、デラビルジン酸塩、リファブチン、エチニルエストラジオール又はノルエチステロンを含む経口避妊薬、フェノバルビタール、フェニトイン、カルバマゼピン、シンバスタチン、アトルバスタチン水和物、タクロリムス、シクロスポリン、エベロリムス、セイヨウオトギリソウ（St. John's Wort、セント・ジョーンズ・ワート）含有食品、アジスロマイシン水和物、

	ボリコナゾール、オメプラゾール、CYP3A4 の基質となる薬剤（シルデナフィルクエン酸塩、タダラフィル、フルチカゾンプロピオン酸エステル、トラゾドン塩酸塩、ルラシドン、クエチアピン、コルヒチン、シロリムス、バルデナフィル、アプレピタント、ホスアプレピタント、イブルチニブ、エバスチン、エリグルスタット、テムシロリムス、ダサチニブ、ダルナビル、トルバプタン、ニソルジピン、フェロジピン、ブデソニド、マラビロク、リルピビリン、ロミタピド等)、サルメテロール、ネビラピン、ボセンタン、メサドン、ロスバスタチン、ワルファリン
解析方法	主解析： 層別ログランク検定
ランダム化の方法	動的割付［割付調整因子：重症度（無症状、軽症）年齢（60 才未満、以上）］
治験実施期間	各実施医療機関の長の許可日～2022 年 3 月 31 日

観察検査スケジュール

	スクリーニング期	治験薬投与期間														経過観察期間			中止時(±1日)
観察/検査/評価項目	同意取得、スクリーニング時	登録時/Day1	Day2	Day3	Day4 (±1日)	Day5	Day6	Day7	Day8 (±1日)	Day9	Day10	Day11 (±1日)	Day12	Day13	Day14 (±1日)	Day19 (±2日)	Day23 (±2日)	Day28 (+7日)	
同意取得	●																		
被験者背景調査	●																		
糖尿病検査	●																		
選択/除外基準確認	●																		
ランダム割付		●																	
COVID-19 の重症度		●																	
治験薬投与		◀━	━	━	━	━	━	━	━	━	━	━	━	━	━▶				
治験薬投与状況確認		●	●	●	●	●	●	●	●	●	●	●	●	●	●				●
食事摂取状況確認		●	●	●	●	●	●	●	●	●	●	●	●	●	●				●
自覚症状		●	●	●	●	●	●	●	●	●	●	●	●	●	●	◀●	●	●▶	●
バイタルサイン		●	○	○	○	○	○	○	○	○	○	○	○	○	○	○	○	○	○
酸素投与		●	●	●	●	●	●	●	●	●	●	●	●	●	●	●	●	●	●
NEWS2 評価		●	○	○	○	○	○	○	○	○	○	○	○	○	○	○	○	○	○
PCR 検査		●	●	●	●	●	●	●	●	●	●	●	●	●	●	◀●	●	●▶	●
血液学的検査	●				○				○			○			○	○			○
血液生化学検査	●				○				○			○			○	○			○
血液凝固検査	●				○				○			○			○	○			○
胸部単純 X 線検査又は胸部 CT 検査	●								○						○				○
併用薬剤・併用療法調査	◀━	━	━	━	━	━	━	━	━	━	━	━	━	━	━	━	━	━	━▶
有害事象調査		◀━	━	━	━	━	━	━	━	━	━	━	━	━	━	━	━	━	━▶
肺炎発症	◀━	━	━	━	━	━	━	━	━	━	━	━	━	━	━	━	━	━	━▶
生存状況	◀━	━	━	━	━	━	━	━	━	━	━	━	━	━	━	━	━	━	━▶

1 背景情報
1.1 はじめに
1.1.1 ネルフィナビルの概略
AG-1343 の有効成分であるメシル酸ネルフィナビルは、米国 Agouron 社において開発された合成抗ヒト免疫不全ウイルス（HIV）薬であり、プラス鎖一本鎖 RNA ウイルスである HIV の複製に関与する HIV アスパラギン酸プロテアーゼを特異的に阻害することにより抗 HIV 活性を発現する。米国では、1997 年 3 月に「HIV 感染症」に対する治療薬として承認された。本邦においては 1998 年 6 月に、「1 回 750 mg を 1 日 3 回」の用法・用量により承認され、2003 年 11 月には投与回数減少による服薬アドヒアランス向上のため「1 回 1250 mg、1 日 2 回」の投与方法が追加となり、2011 年 3 月には再審査結果が通知され、承認された効能・効果、用法・用量で有効性・安全性が認められると判断されたが、HIV 治療薬の変遷により、2019 年 11 月に経過措置医薬品として告示され、2020 年 3 月 31 日に薬価基準からは削除された。

1.1.2 新型コロナウイルス感染症（COVID-19）の病態
COVID-19 は、重症呼吸器症候群コロナウイルス 2（SARS-CoV-2）による新興感染症である。2019 年 12 月中国湖北省武漢市の原因不明の肺炎の集団発生から始まり [1)]、世界中に感染が拡大し 2020 年 3 月 11 日には世界保健機関から「国際的に懸念される公衆衛生上の緊急事態」であると宣言され、2020 年 4 月 30 日現在 200 を超える国又は地域に 300 万人以上の感染者数、20 万人以上の死亡者数が報告されている。

コロナウイルス科は、直径 80～160 nm のエンベロープを有するプラス鎖一本鎖リボ核酸（RNA）ウイルスである。遺伝学的特徴から α、β、γ、及び δ の属に分類され、ヒトに主に風邪症候群を起こすのは α 及び β 属である。重篤な感染症を引き起こした SARS コロナウイルス及び MERS（Middle East Respiratory Syndrome）コロナウイルスと同様 SARS-CoV-2 は同じ β 属である [2,3)]。

中国からの報告では、COVID-19 患者の年齢中央値は 47 歳（四分位範囲 35.0-58.0）でやや男性に多く（58.1%）、潜伏期間は 4 日（中央値、四分位範囲 2-7）であり、主な症状は発熱（43.8%）、倦怠感（38.1%）、呼吸器症状［咳嗽（67.8%）、喀痰（33.7%）、呼吸困難（18.7%）］、多くはないものの筋肉痛又は関節痛（14.9%）、消化器症状［嘔気又は嘔吐（5.0%）、下痢（3.8%）］等も認められている [4)]。2020 年 3 月 3 日の時点での世界保健機関による推定死亡率は 3.4%と発表されており [5)]、重症例は高齢者、基礎疾患を有する症例に多い傾向が認められている [4,6)]。

1.1.3 COVID-19 の診断、治療
COVID-19 に比較的多い症状としては長く続く発熱や呼吸器症状であるが、特異的な症状や所見はない。血液検査でも特異的な所見はないが、COVID-19 は肺炎の合併頻度が高いため、胸部単純 X 線や胸部 CT 検査は、斑状陰影、すりガラス陰影や浸潤影の検出に有用である。これら臨床的特徴と疫学的な背景に加え、上下気道由来検体からのウイルス分離又はポリメラーゼ連鎖反応（PCR）による SARS-CoV-2 の遺伝子検出により診断が確定される。

COVID-19 の軽症・中等症に対して現時点では抗ウイルス薬による特異的な治療法は確立されていないため対症療法が中心となり、重症では抗ウイルス薬であるレムデシビルの投与、人工呼吸管理や体外式膜型人工肺（ECMO）等を含めた集中治療が必要となる。抗ウイルス薬については、抗 HIV 薬のロピナビル・リトナビル配合剤、抗インフルエンザ薬のファビピラビル、喘息治療薬のシクレソニド、レムデシビル、抗マラリア薬のヒドロキシクロロキン等が抗ウイルス効果を期待され、様々な重症度に対し臨床試験が行われている。

1.2 治験に関する背景情報
1.2.1 背景
COVID-19 パンデミックの状況下、ファビピラビル、レムデシビル、シクレソニド、ロピナビル・リトナビル、クロロキン、ヒドロキシクロロキン等が COVID-19 に対する抗ウイルス薬として世界各国で様々な重症度に対する臨床試験が行われている中 [7-11)]、レムデシビルは米国立衛生研究所が実施する臨床試験の予備的解析において重症 COVID-19 による肺炎で入院した患者の回復までの期間がプラセボに比べ 31%有意に速かったという結果を受け、米国食品医薬品局において重症の COVID-19 患者に対しての緊急使用が 2020 年 5 月 1 日に許可され、本邦においても 2020 年 5 月 7 日に重症の COVID-19 患者に対し特例承認がなされた。COVID-19 に対する抗ウイルス薬については、重症の COVID-19 患者に対す

るレムデシビルが薬事承認を受けているのみと極めて限られており、また軽症や中等症に対する高いエビデンスレベルで有効性及び安全性が確認された薬剤はない。

新興感染症に対する新規治療薬やワクチンを開発するためには多くの時間が必要であることから、既承認薬を利用することにより、迅速に治療法を確立することは極めて重要である。Ohashi ら研究グループは、25 研究機関の共同研究において、既に何らかの疾患に対して欧州医薬品庁／米国食品医薬品局／医薬品医療機器総合機構から承認を受けている 306 の薬剤について、Vero E6/TMPRSS2 発現細胞を用いてインビトロの SARS-CoV-2 増殖への効果を検証した結果、抗 HIV 薬であるネルフィナビル及び白血球減少症等で使用されるセファランチンが高いウイルス増殖に対する細胞障害抑制効果があることを報告した [12)]。この中では、インシリコにおける薬剤ドッキングシミュレーションによる作用機序が検討され、ネルフィナビルは SARS-CoV-2 複製に必須のメインプロテアーゼに、セファランチンはウイルスと細胞の吸着に必要なウイルススパイクタンパク質にそれぞれ結合することで作用することが示され、さらに抗ウイルス効果の数理解析の予測では各薬剤単独だけでなく併用による相乗効果もあることが示された。ウイルス増殖抑制に直接的に効果が期待できるネルフィナビル、ウイルスの細胞接着を抑制することで効果が期待できるセファランチン、両薬剤とも本邦で使用可能な薬剤であり、COVID-19 に対する抗ウイルス薬として高い有効性が期待できる薬剤であると考えている。

1.2.2 被験薬選択の根拠

COVID-19 に対する有効性及び安全性が示された抗ウイルス薬が極めて限られている状況において、治療効果の期待できる薬剤を探索することは喫緊の課題である。ネルフィナビルは COVID-19 患者に対する投与経験はないものの、Ohashi らの報告から、インビトロにおいて現在臨床試験が実施されているロピナビル及びクロロキンより高い抗ウイルス効果が期待できること、インシリコにおいて SARS-CoV-2 に対し直接的にウイルス増殖抑制効果が期待でき、且つ COVID-19 患者の重症度の違いによるウイルス量動態変化の報告に基づくシミュレーションから HIV 治療に対するネルフィナビルの用法・用量においてウイルス排除期間の短縮が期待できることから、COVID-19 患者に対しても有効であることが大いに期待される薬剤であることから被験薬として選択した [12,13)]。

また、ネルフィナビルは HIV に対するキードラッグとして、核酸系逆転写酵素阻害剤 2 剤と併用して使用する HIV 治療薬であり、現在では新規作用機序の薬剤の上市により抗 HIV 治療ガイドラインには記載されておらず使用は極めて限られていると考えられるものの、その有効性及び安全性は十分に検証され、併用薬の多い日本人 HIV 患者における使用成績調査でも副作用発現率 57.9%（655/1,132 例）と治験時データからの増加はなく、副作用のプロファイルについても十分な期間評価がなされているため、COVID-19 患者に対しても忍容性については許容可能な範囲であるものと考えている。

1.2.3 本治験計画の根拠

COVID-19 に対するネルフィナビルの抗ウイルス効果、臨床的有効性、及び安全性を検討することを目的として、無症状及び軽症の COVID-19 は治療法が確立していないことから対症療法を対照群と設定し、COVID-19 患者に対する投与経験がないため無症状及び軽症 COVID-19 患者を対象として、ランダム化非盲検並行群間比較試験を立案した。

1.2.4 海外での臨床試験及び開発状況

当該疾患に対して、ネルフィナビルは海外で臨床試験は実施されていない。

1.2.5 国内での臨床試験及び開発状況

当該疾患に対して、ネルフィナビルの国内で臨床試験は実施されていない。

1.2.6 本治験の妥当性

新興感染症である COVID-19 に対する確立された治療法は極めて限られており、感染拡大が続いている現状において、治療効果の期待できる薬剤を探索することは喫緊の課題である。HIV に対し既承認であるネルフィナビルは、COVID-19 患者に対する投与経験はないものの、インビトロ及びインシリコにおける COVID-19 に対する抗ウイルス効果、COVID-19 のウイルス量動態変化に戻づくシミュレーションの結果から、既に HIV 治療に用いられる用法・用量において優れた抗ウイルス効果が期待できる可能性が高いものと考えることから、対症療法を対照群としてランダム化非盲検並行群間比較試験を立案した。

安全性については、日本人 HIV 患者に対しても投与経験があり、2011 年 7 月には再審査結果が通知され、安全性について特段の問題はないと判断され、副作用プロファイルもある程度予測できるため事前に適切な対応体制の構築が可能であることから、COVID-19 患者に対しても安全性を担保しつつ治験を実施することは可能であると考えている。

本治験は、無症状及び軽症 COVID-19 患者を対象とするが、無症状及び軽症者においても早期にウイルス陰性化を図ることができれば、重症化抑制や感染拡大の防止にも寄与するものであり、治療法確立及び公衆衛生の観点からも意義のある研究であると考えている。

以上のことから、無症状及び軽症 COVID-19 患者を対象として、ネルフィナビルの抗ウイルス効果、臨床的有効性、及び安全性を検討する治験を実施することは妥当であると判断した。

1.2.7 医薬品の臨床試験の実施の基準に関する省令（GCP）遵守に関する陳述

本治験は「ヘルシンキ宣言（2013 年 10 月改訂）」、治験実施計画書、薬機法第 14 条第 3 項及び第 80 条の 2 に規定する基準、並びに GCP を遵守して実施する。

2 治験の目的

無症状及び軽症 COVID-19 患者において、被験薬投与群及び対症療法群における SARS-CoV-2 ウイルス量の変化、臨床症状の改善、有害事象発現を評価し、ネルフィナビルの COVID-19 に対する抗ウイルス効果、臨床的有効性及び安全性を評価することを目的とする。

3 治験方法

3.1 治験のデザイン

本試験は、医師主導、多施設共同ランダム化非盲検並行群間比較試験で実施する。

3.1.1 治験デザイン設定の根拠

COVID-19 に対し有効性及び安全性を検証された抗ウイルス薬は極めて限られている。本治験は、早期に抗ウイルス効果、臨床的有効性及び安全性を探索することを目的とし、多施設共同、対症療法を対照群とした、ランダム化非盲検並行群間比較試験として設定した。現時点における軽症、中等症 COVID-19 患者に対する確立された治療法はなく、症状に応じた対症療法を行うことが基本であるため、対症療法群を対照群として設定することに倫理的な問題はないものと考えている。

3.2 対象疾患

無症状及び軽症 COVID-19 を対象疾患とする。

3.2.1 対象集団選択の根拠

本治験においては、無症状及び軽症の COVID-19 患者を対象とする。

無症状及び軽症の定義は以下のとおりである。

- 無症状患者：上下気道検体から PCR 検査等において SARS-CoV-2 が検出された患者で、且つ COVID-19 によると判断される臨床症状を認めない患者
- 軽症患者：上下気道検体から PCR 検査等において SARS-CoV-2 が検出された患者で、37.0℃以上の発熱、咳嗽、呼吸困難等の何らかの COVID-19 によると判断される臨床症状を認める患者

AG-1343 はインビトロ及びインシリコにおける検討、COVID-19 患者の重症度の違いによるウイルス量動態変化の報告に基づいたシミュレーションの結果、及び抗 HIV 薬としてのネルフィナビルの使用経験から、既に HIV 治療に用いられる用法・用量において SARS-CoV-2 に対する抗ウイルス効果が期待でき、安全性についても懸念が少ないものであると考える[12,13)]。しかし COVID-19 患者に対する使用経験はないため、酸素投与や人工呼吸管理等が必要である重症度の高い患者を対象として被験薬を投与し予期せぬ有害事象の発現によりさらなる重症化を来す懸念が否定できず、安全性を十分に確保した状況で治験を実施することが困難である可能性があるため、無症状及び軽症 COVID-19 患者を対象とする。また、無症状患者については経過観察が基本ではあるものの、抗ウイルス効果の評価は可

能であり、また無症状患者の約13%は臨床症状が出現していることから、ウイルス量減少の評価だけでなく、発症抑制についても検討するため、対象患者とした[14)]。

重症度については、明確に定義されたものはなく、米国胸部学会市中肺炎の重症度基準、肺炎／呼吸不全／多臓器不全等の有無での分類等が行われているが、本研究においては、安全性の観点から呼吸状態の安定している、酸素投与の必要のない症状のある患者を軽症患者、症状のない患者を無症状患者として定義することとした[4,15)]。

3.3 目標被験者数・治験薬投与期間

3.3.1 目標被験者数・治験薬投与期間

・目標被験者数：120 例

被験薬投与群：60 例

対症療法群：60 例

・治験薬投与期間：14 日間又は 15 日間

3.3.2 目標被験者数の設定根拠

既報の感染者体内でのウイルス動態（viral load）データ[16-20)]から nonlinear mixed effect model を用いてウイルス動態パラメータ（*the rate constant for virus infection*, *the death rate of infected cells*, *the maximum viral replication rate for coronavirus infections*, *the initial number of uninfected target cells*）[21)]を推定した。viral RNA load の初期値を 100 copies/mL、初期状態からランダム化（Day1）までの日数が期待値 4 の指数分布に従うものとし、推定されたウイルス動態パラメータの確率分布より、ウイルス量が 0 になるまでの日数（陰性化日数）のシミュレーションデータを生成した。（サンプルサイズ：2000、被験薬投与群、対症療法群それぞれにつき 1000 ずつ。）このシミュレーションデータに対して陰性化日数 ＞ 28.0 を右側打ち切りとした解析により、ウイルス量が 0 になる日をイベント発生とした割り付け群のハザード比は、1.79 と推定された。このハザード比のもとで帰無仮説：$S(t|z=1)=S(t|z=0)$（ただし S は生存時間関数、t は時間、z は割り付け群）に対するログランク検定（有意水準両側 5%）で検出力 80%を得るのに必要なイベント数は 97.345 であった。さらに、この必要イベント数をシミュレーションデータに占める右側打ち切りを起こすデータの割合（0.096）で除した数値(107.7)、および同意撤回等による脱落を加味し、目標症例数は 120 例と設定した。

3.4 評価項目

3.4.1 主要評価項目

登録時からウイルス陰性化までの日数とする。

【評価方法】

登録時を Day1 として、Day1 から 28 日後（Day28）までを観察期間として、2 回の唾液 PCR 検査にて連続して「陰性」と判定されるとき「ウイルス陰性化」とし、これらの 2 回の唾液 PCR 検査のうち 1 回目に「陰性」となった日をウイルス陰性化の日として、ウイルス陰性化の日までの日数を評価する。（唾液 PCR 検査の測定および「陰性」判定については「6.3 試料の採取及び測定」を参照）

3.4.1.1 主要評価項目の設定根拠

本治験は、AG-1343 がインビトロ及びインシリコの検討において SARS-CoV-2 に対し優れた抗ウイルス効果が期待できることが明らかとなった報告に基づき、AG-1343 の COVID-19 に対する抗ウイルス効果、並びに臨床的有効性及び安全性を探索する臨床試験として設定している[12)]。ウイルス量の減少と臨床症状の改善との関連性は明らかになっていないものの、本治験は探索的試験であり、臨床症状の有効性評価に確立された評価方法はないことから、本治験においては定量評価が可能であるウイルス量について、陰性化までの日数を主要評価項目として設定した。

3.4.2 副次評価項目

副次評価項目は以下の有効性及び安全性評価項目とする。

● 有効性評価項目

＜共通項目＞

(1) ウイルス量の曲線下面積：ウイルス量の曲線下面積は、登録時から 28 日間における唾液 PCR

検査によるウイルス量より算出する。
(2) ウイルス量の半減期：ウイルス量の半減期は、登録時から 28 日間における唾液 PCR 検査によるウイルス量より算出する。
(3) 各時点における体温
(4) 全死亡者の割合：全死亡者の割合は、登録時から 28 日間での全死因による死亡の有無より算出する。
(5) 肺炎発症率：肺炎発症率は、登録時から肺炎発症までの日数より算出する。
(6) 肺炎発症者の割合：肺炎発症者の割合は、登録時から 28 日後までの COVID-19 によると判断される肺炎の発症の有無より算出する。
(7) 酸素投与率：酸素投与率は、登録時から「24 時間以上持続する酸素投与」の開始までの日数より算出する。
(8) 酸素投与者の割合：酸素投与者の割合は、登録時から 28 日後までの「24 時間以上持続する酸素投与」の開始の有無より算出する。

＜無症状者＞
(1) COVID-19 発症者の割合：COVID-19 発症者の割合は、登録時から 28 日間での COVID-19 によると判断される「2 日連続する 37.0℃以上の発熱」、「24 時間以上持続する咳嗽」を含め何れかの症状の発現の有無より算出する。
(2) 発熱症状を発現した被験者の割合：発熱症状を発現した被験者の割合は、登録時から 28 日間での「2 日連続する 37.0℃以上の発熱」の発現の有無より算出する。
(3) 咳嗽症状を発現した被験者の割合：咳嗽症状を発現した被験者の割合は、登録時から 28 日間での「24 時間以上持続する咳嗽」の発現の有無より算出する。

＜軽症者＞
(1) 解熱率：解熱率は、登録時から体温 37.0℃未満に回復するまでの日数より算出する。
(2) 臨床症状の全消失率：臨床症状の全消失率は、COVID-19 によると判断される全ての臨床症状が消失するまでの日数より算出する。
(3) 症状ごとの改善率：症状ごとの改善率は、COVID-19 によると判断される臨床症状が改善するまでの日数（症状の内容ごとに評価）より算出する。

肺炎の定義：胸部単純 X 線又は胸部 CT において肺炎像が出現した場合
COVID-19 発症の定義：唾液 PCR 検査が陽性であり、且つ COVID-19 によると判断される何らかの臨床症状が発現した場合
「24 時間以上持続する咳嗽」の定義：症状日誌（付録 2）にて咳嗽について「あり」が連続して 2 回以上続いているとき、そのうち 1 回目の記録を「24 時間以上持続する咳嗽」:「あり」とする。
症状改善の定義：体温 37.0℃未満、SpO_2≧96%（室内気）、その他の症状は症状が 5 割以上軽減した状態が 48 時間以上持続した場合

- 安全性評価項目
(1) 有害事象（有害事象発現割合、重篤な有害事象発現割合、副作用発現割合、重篤な副作用発現割合、）
(2) 臨床検査（血液学的検査、血液生化学検査、バイタルサイン）

3.4.2.1 副次評価項目の設定根拠

COVID-19 に対する臨床的有効性及び安全性を評価するガイダンスやガイドライン等はない。本治験では副次評価項目では、安全性評価のために有害事象及び臨床検査を評価項目として設定した。また、有効性評価については、臨床的有効性として、ウイルス量早期減少、発症抑制、臨床症状改善時間短縮、重症化抑制を評価することを目的として、無症状者及び軽症者それぞれについて、ウイルス量変化、体温、COVID-19 によると判断される臨床症状、全死亡、酸素投与及び肺炎合併の項目を設定した。

3.4.3 探索的項目

有効性の探索的項目として、NEWS2（National Early Warning Score）の収集を行う [22]。NEWS2 は、急性期疾患の状態悪化の早期検出及び早期対応を行うためのスコアリングシステムであり、COVID-19 の

臨床研究でも用いられているものである。本治験においても、軽症者の重症化早期検出の可能性を評価するため、探索的項目として収集する。

NEWS2

項目／スコア	3	2	1	0	1	2	3
呼吸数	8 以下	-	9-11	12-20	-	21-24	25 以上
SpO2	91 以下	92-93	94-95	96 以上	-	-	-
酸素投与	-	あり	-	なし	-	-	-
体温	35.0 以下	-	35.1-36.0	36.1-38.0	38.1-39.0	39.1 以上	-
収縮期血圧	90 以下	91-100	101-110	111-219	-	-	220 以上
脈拍数	40 以下	-	41-50	51-90	91-110	111-130	131 以上
意識状態	-	-	-	清明	-	-	混濁、呼びかけに反応、痛み刺激に反応、無反応

3.5 治験の手順

3.5.1 被験者の選定からスクリーニング期までの手順

(1) 被験者の選定

治験責任医師又は治験分担医師は、患者の全身状態、原疾患及び合併症の重症度・症状、年齢、同意能力、他の治験参加の有無等を考慮し、患者が本治験の対象として適格であるか否かを検討する。

(2) 同意取得

治験責任医師又は治験分担医師は、本治験の対象として適切と判断した患者に対して説明を十分に行い、文書にて同意を取得する（「11.1.3 同意取得の時期と方法」参照）。

(3) スクリーニング期

治験責任医師又は治験分担医師は各実施医療機関における同意取得順に被験者識別コードを割り当て、被験者スクリーニング名簿に記載する。被験者識別コードを割り当てた後、「6.2 観察・検査スケジュール」の記載に基づきスクリーニング時に規定した観察・検査を実施する。

3.5.2 被験者の適格性判定及び登録の手順

(1) 適格性の判定

治験責任医師又は治験分担医師は、スクリーニング時および登録時/Day1 の検査結果を含めて選択基準及び除外基準に基づき、被験者の適格性を判定する。

(2) 登録

適格性判定の結果、適格性が確認された者は被験者として登録される。重症度の判断は登録時点で実施する。被験者は被験薬投与群、若しくは対症療法群のうちいずれかの群にランダムに割付けられる。(割付調整因子を重症度（無症状、軽症)、年齢（60 歳未満、以上）とした動的割り付け)

3.5.3 登録以降の手順

(1) 治験薬初回投与

被験薬投与群には、原則として、ランダム割付同日から治験薬の初回投与を開始する。対症療法群には治験薬の投与は行わず、ランダム割付同日を登録時/Day1 と見なして以降の規定の検査を実施する。

(2) 治験薬服用期間

治験薬服用期間は 14 日間又は 15 日間（合計 42 回分の投与）とする。

(3) 経過観察期間

治験薬服用期間（14 日間又は 15 日間）が終了した後、経過観察期間に移行し規定の検査を実施する。

(4) 各被験者の治験中止

中止基準を満たした場合は、治験薬服用を中止し規定の検査を実施する。治験を中止した場合、中

止の登録を行う。

3.6 各被験者の治験期間

各被験者の治験期間は、同意取得日から経過観察期間の最終観察日又は中止検査時のいずれか遅い時点までとする。各被験者の治験参加予定期間は、治験薬投与期間である 14 日間又は 15 日間、経過観察期間である 14 日間の合計 28 日間である。

3.7 治験実施予定期間

各実施医療機関の長の許可日～2022 年 3 月 31 日

4 被験者の選択、除外、中止基準

4.1 選択基準

無症状及び軽症 COVID-19 患者で、以下のすべての基準を満たす場合に登録可とする。

(1) 同意取得時に 20 歳以上の日本人患者、男女の性別は問わない

(2) 同意取得前 3 日以内に上下気道由来検体から PCR 検査又は LAMP 法で SARS-CoV-2 が検出された患者、又は抗原検査が陽性であった患者

(3) 説明文書の内容、その他、治験に関する事項について十分な説明を受け、その内容を理解し、本治験への参加について自由意思に基づく文書による同意が得られた患者

＜設定理由＞

(1) 患者の多くは成人であること、小児における被験薬の投与経験は限られているため 20 歳以上と設定した。

(2) COVID-19 診断確定患者を対象とするため設定した。

(3) GCP 省令及びヘルシンキ宣言の精神を遵守し設定した。

4.2 除外基準

以下のうち一つでも該当する患者は対象から除外する。

(1) 登録時点で症状出現から 8 日以上経過している患者

(2) 経皮酸素飽和度（SpO_2）が 96%未満（室内気）の患者

(3) スクリーニング時検査において以下のいずれかに該当する患者

1) ALT 又は AST が基準値上限の 5 倍を超える
2) Child-Pugh 分類 B 又は C に該当する
3) 血清クレアチニンが基準値上限の 2 倍を超え、かつクレアチニンクリアランスが 30 mL/min 未満*の場合

*Cockcroft-Gault 式による推定クレアチニンクリアランス値。ただし実測値がある場合は実測値を用いる。

(4) コントロール不良の糖尿病患者（糖尿病の治療を受けているにもかかわらず、随時血糖が 200 mg/dL 以上又は HbA1c が 7.0 %以上の糖尿病患者）

(5) 重篤な疾患を合併しており、治験対象として治験責任医師又は治験分担医師が不適当と判断した患者

(6) 血友病患者又は著しい出血傾向のある患者

(7) 高度な下痢症状がある患者

(8) 被験薬の成分に対し過敏症の既往歴のある患者

(9) 授乳中、妊娠中、又は妊娠の可能性がある女性患者

(10) 被験薬初回投与日から治験参加の期間、適切な避妊法を用いることができない患者（男性及び女性は妊娠の可能性がある女性）

(11) リファンピシンを同意取得前 2 週間以内に服用した患者

(12) 同意取得前 12 週以内に他の治験又は臨床試験に参加し投薬を受けた患者

(13) HIV 感染症治療中の患者

(14) 新型コロナワクチンの接種歴のある患者、治験参加中に新型コロナワクチン接種を希望する患者

(15) その他、治験責任医師あるいは治験分担医師が不適当と判断した患者

＜設定理由＞

(1) ：症状出現から長時間経過している場合、抗ウイルス効果が期待できない可能性が高いため設定した。

(2) ：本治験は、軽症 COVID-19 患者を対象とするため設定した。

(3) 1) 2) (4) (6)～(8)：治験薬の特性から、被験者の安全性を確保するため設定した。

(3) 3) (5) (11) (12) (14)：被験薬の有効性及び安全性を適切に評価するため設定した。

(9) (10)：被験薬の妊婦及び胎児への安全性を懸念し設定した。

(13) ：ネルフィナビル投与により薬剤耐性 HIV が発現する可能性があるため設定した。

(15) ：被験者の安全性を確保し、適正に本治験を実施する上で、不適格な被験者を治験責任医師あるいは治験分担医師が除外できる余地を残すため設定した。

4.3 中止基準

4.3.1 被験者の治験の打ち切り

治験責任医師等は下記の基準に該当した場合は当該被験者について治験を中止する。

(1) 被験者が同意を撤回した場合

(2) 生命を脅かす：緊急処置を要する、有害事象が発現した場合

(3) COVID-19 の悪化により、治験責任医師又は治験分担医師が治験の継続が困難又は適切でないと判断された場合

(4) その他治験責任医師、治験分担医師、又は効果安全性評価委員会により治験の継続が困難又は適切でないと判断された場合

4.3.2 中止時の対処

治験責任医師等は、当該被験者について治験を中止する際には必要に応じて被験者へ説明し、説明内容及び被験者の反応について記録する。

被験者からの協力が得られた場合は、中止時検査を実施し、Day28 までの生存状況を確認する。

中止後の被験者の治療については、被験者の不利益とならないように対応する。

5 治験薬

5.1 被験薬及び対照薬

(1) 被験薬（治験薬概要書参照）

名称	AG-1343 錠 250 mg
製造元	日本たばこ産業株式会社、ファイザー株式会社
含有量・剤型	1 錠中ネルフィナビル 250 mg（ネルフィナビルメシル酸塩として 292.25mg）を含有するうすい青色のフィルムコーティング錠
添加物	クロスポビドン、ケイ酸カルシウム、ステアリン酸マグネシウム、青色 2 号、ヒプロメロース、トリアセチン
貯法	各包装ラベルに従う
分子式	$C_{32}H_{45}N_3O_4S$・ CH_4O_3S
分子量	663.89
作用機序	HIV プロテアーゼ阻害薬（サキナビルタイプ）

(2) 対照薬

本治験では対照薬は設定しない。

5.2 包装及び表示

治験調整医師は、以下のように包装、表示した被験薬、およびラベル貼替用シールを実施医療機関の治験薬管理者に交付する。

製造元	日本たばこ産業株式会社	ファイザー株式会社
包装形態	300 錠を 1 ボトルとする。	126 錠を 1 ボトルとする。

表示	ボトルに貼付されているラベルは以下の内容を含むものとする。<br>1) 製造番号<br>2) 使用期限	ボトルに貼付されているラベルは治験用と表示し以下の内容を含むものとする。<br>1) 治験薬記号<br>2) 製造番号<br>3) 使用期限<br>4) 治験実施計画書番号<br>5) 貯蔵方法<br>6) 治験調整医師：住所/職名/氏名
ラベル貼替用シール	ラベル貼替用シールは治験用と表示し、以下の内容を含むものとする。<br>1) 治験薬記号<br>2) 治験実施計画書番号<br>3) 貯蔵方法<br>4) 治験調整医師：住所/職名/氏名	なし

5.3 治験薬の保管・管理手順

実施医療機関に納入された治験薬は、治験薬管理者が管理する。残余治験薬、使用済み治験薬・包装箱は治験薬管理者が管理し、残余治験薬・包装箱は実施医療機関にて廃棄する。これらに係る手順については、治験調整医師により提供する「治験薬の管理に関する標準業務手順書」に従う。

5.4 その他、治験薬以外の実施医療機関への提供物

・中央測定用試料採取キット

6 被験者に対する治療

6.1 治験薬の投与

6.1.1 期間及び用法・用量

(1) スクリーニング期

治験薬の投与は行わない。

(2) 治験薬投与期間

被験薬投与群には、ランダム化当日から 14 日間又は 15 日間、1 日 3 回食後に治験薬の投与（合計 42 回）を行う。（ただし、2 回連続で唾液 PCR 陰性が確認された場合、治験責任医師又は治験分担医師の判断で、治験薬服用は終了可能とする）対症療法群には治験薬の投与は行わない。

(3) 経過観察期間

治験薬の投与は行わない。

6.1.1.1 治験期間の設定根拠

治験薬の投与期間を 14 日間と設定することから、その後の COVID-19 に関連する症状の経過及び有害事象発現の観察期間を 14 日間と設定し、治験期間は 28 日間と設定する。

COVID-19 は、感染曝露から発症までの潜伏期間の中央値は 5.1 日（95%信頼区間：4.5-5.8 日）であり、発症者の 97.5%は 11.5 日以内に発症すると推定されるという報告があるため、本治験設定期間では、無症状者における発症までの経過を十分評価できるものであると考える [23)]。

6.1.2 投与方法

ネルフィナビルとして 1 回 750 mg を 1 日 3 回経口投与する。食後の内服を原則とするが、食事摂取困難な場合は、食事摂取に相当する時間に投与する。投与期間は 14 日間又は 15 日間とし、ランダム割付同日に治験薬投与を開始する（合計 42 回分の投与）。

6.1.2.1 被験薬の用法・用量の設定根拠

COVID-19 患者の重症度の違いによるウイルス量動態変化の報告及び Ohashi らのネルフィナビ

ルの抗ウイルス効果に基づいたシミュレーション結果から、HIV 感染症に対する用法・用量である 1 回 1,250 mg を 1 日 2 回、又は 1 回 750 mg を 1 日 3 回いずれの用法・用量においても SARS-CoV-2 に対する抗ウイルス効果が期待できることが予測された[12,13)]。HIV に対する抗ウイルス効果としては、より高いトラフ濃度を維持することが重要であることも考慮し、本治験における用法・用量は 1 回 750 mg を 1 日 3 回食後に経口投与を選択した。尚、本用法・用量は、日本人 HIV 患者に対する安全性が評価されており、副作用プロファイルもある程度予測可能であるため、リスクを最小化した下での治験実施が可能であると考えている。

6.1.3 被験薬の休薬基準

下記の基準に従って被験薬の休薬を行う。

- 被験薬と因果関係があると治験責任医師又は治験分担医師が判断し、高度又は医学的に重大、若しくは許容できないと判断した有害事象が認められた場合
- その他、安全性上問題があると治験責任医師又は治験分担医師が判断した場合

6.1.4 被験薬の中止基準

下記の基準に従って被験薬の投与を中止する。

- 治験責任医師又は治験分担医師が治験薬と因果関係があると判断した、高度又は医学的に重大である有害事象が、治験薬の休薬後 4 日以内に中等度以下まで回復しない場合
- その他、安全性上問題があると治験責任医師又は治験分担医師が判断した場合

6.2 観察・検査スケジュール

「治験スケジュール」（表 1）に従い、診察、検査を行い情報を収集する。唾液 PCR 検査については測定を国立感染症研究所に委託し、他は各実施医療機関にて実施する。また、唾液 PCR 検査及び自覚症状調査は、登録時/Day1 から Day28 まで毎日実施する。ただし、3.4.1 主要評価項目に定義するウイルス陰性化確認以降は唾液 PCR の実施は不要とする。

治験薬投与期間以後の観察・検査は登録時/Day1 を起点としたスケジュールで行う。Day4/8/11/14/中止時の血液学的検査・血液生化学的検査・血液凝固検査の実施は±1 日、Day19/23 の観察・検査は±2 日、Day28 の観察・検査は＋7 日を許容範囲として実施する。

また、治験責任医師又は治験分担医師が必要と判断した場合には規定した実施時期以外に必要な項目について観察・検査を行う。

6.2.1 治験スケジュール

表 1

	スクリーニング期	治験薬投与期間														経過観察期間			中止時(±1 日)
観察/検査/評価項目	同意取得、スクリーニング時	登録時/Day1	Day2	Day3	Day4 (±1 日)	Day5	Day6	Day7	Day8 (±1 日)	Day9	Day10	Day11 (±1 日)	Day12	Day13	Day14 (±1 日)	Day19 (±2 日)	Day23 (±2 日)	Day28 (+7 日)	
同意取得	●																		
被験者背景調査	●																		
糖尿病検査	●																		
選択/除外基準確認	●																		
ランダム割付		●																	
COVID-19 の重症度		●																	
治験薬投与		←	—	—	—	—	—	—	—	—	—	—	—	—	→				
治験薬投与状況確認		●	●	●	●	●	●	●	●	●	●	●	●	●	●				●
食事摂取状況確認		●	●	●	●	●	●	●	●	●	●	●	●	●	●				●
自覚症状		●	●	●	●	●	●	●	●	●	●	●	●	●	●	←●	●	●→	●
バイタルサイン		●	○	○	○	○	○	○	○	○	○	○	○	○	○	○	○	○	○
酸素投与		●	●	●	●	●	●	●	●	●	●	●	●	●	●	●	●	●	●
NEWS2 評価		●	○	○	○	○	○	○	○	○	○	○	○	○	○	○	○	○	○
PCR 検査		●	●	●	●	●	●	●	●	●	●	●	●	●	●	←●	●	●→	●
血液学的検査	●				○				○			○			○	○			○
血液生化学検査	●				○				○			○			○	○			○
血液凝固検査	●				○				○			○			○	○			○
胸部単純 X 線検査又は胸部 CT 検査	●								○						○				○
併用薬剤・併用療法調査	←	—	—	—	—	—	—	—	—	—	—	—	—	—	—	—	—	—	→
有害事象調査		←	—	—	—	—	—	—	—	—	—	—	—	—	—	—	—	—	→
肺炎発症	←	—	—	—	—	—	—	—	—	—	—	—	—	—	—	—	—	—	→
生存状況	←	—	—	—	—	—	—	—	—	—	—	—	—	—	—	—	—	—	→

6.2.2 観察・検査項目

スクリーニング期の観察・検査項目について、同意取得前 1 日以内に観察・検査を行っている場合には、同意取得前直近のデータを採用できる。

(1) 被験者背景調査

観察項目：生年月日、性別、既往歴、合併症、罹病期間（症状出現からランダム化までの時間）、SARS-CoV-2 検査方法（PCR 検査・LAMP 法、抗原検査）

観察時期：同意取得、スクリーニング時

(2) 糖尿病検査

観察項目：HbA1c

観察時期：同意取得、スクリーニング時

(3) COVID-19 の重症度

観察項目：COVID-19 の重症度（無症状/軽症）

観察方法：同意取得後～ランダム化前直近の自覚症状および体温を確認し、自覚症状無しかつ体温 37.0℃未満であれば無症状、自覚症状ありまたは体温 37.0℃以上であれば軽症とする。また、胸部単純 X 線写真又は胸部 CT にて肺炎像が認められた場合でも、自覚症状が無ければ無症状とする。

観察時期：登録時/Day1

(4) 治験薬投与状況確認

観察項目：投与状況の確認

観察方法：登録時/Day1 に被験者へ服薬日誌（付録 1）を交付の上、記録に関して説明し、服薬日誌や服用後の残薬等の目視によって確認する。

観察時期：登録時/Day1、Day2、Day3、Day4、Day5、Day6、Day7、Day8、Day9、Day10、Day11、Day12、Day13、Day14、（必要時 Day15）、中止時

(5) 食事摂取状況確認

観察項目：食事摂取状況の確認

観察方法：登録時/Day1 に被験者へ服薬日誌（付録 1）を交付の上、記録に関して説明し、治験薬服用時の食事状況の記録によって確認する。

観察時期：登録時/Day1、Day2、Day3、Day4、Day5、Day6、Day7、Day8、Day9、Day10、Day11、Day12、Day13、Day14、（必要時 Day15）、中止時

(6) 自覚症状

観察項目：咳嗽、呼吸困難、喀痰、鼻汁、鼻閉、咽頭痛、倦怠感、頭痛、食欲不振、下痢、嘔気、嘔吐、関節痛、筋肉痛、味覚異常、嗅覚異常、その他

観察方法：登録時/Day1 に被験者へ症状日誌（付録 2）を交付の上、記録に関して説明し、自覚症状の記録によって確認する。登録時/Day1 の記録は医師と共に確認し記載する。

観察時期：登録時/Day1～Day28 まで連日

(7) バイタルサイン

観察項目：血圧、脈拍数、体温、SpO_2、呼吸数、意識状態

観察方法：体温は被験者療養場所（自宅など）でも可能な限り測定を実施して、一日の最高体温を症状日誌（付録 2）へ記録する。ただし登録時/Day1 の体温は登録時点での体温を記録する。SpO_2 は、一日の最低値を記録する。意識状態は、清明もしくは清明ではない（混濁、呼びかけに反応、痛み刺激に反応、無反応）かを判断し記録する。

観察時期：登録時/Day1、Day2、Day3、Day4、Day5、Day6、Day7、Day8、Day9、Day10、Day11、Day12、Day13、Day14、Day19、Day23、Day28、中止時

（ただし、登録時/Day1 以外の観察時期は、入院/外来の状況を考慮して可能な限り実施する）

(8) 酸素投与

観察項目：酸素投与
観察方法：酸素投与の分時流量を記録する
観察時期：登録時/Day1、Day2、Day3、Day4、Day5、Day6、Day7、Day8、Day9、Day10、Day11、Day12、Day13、Day14、Day19、Day23、Day28、中止時

(9) NEWS2 評価

観察項目：NEWS2
観察方法：3.4.3 項 参照
観察時期：登録時/Day1、Day2、Day3、Day4、Day5、Day6、Day7、Day8、Day9、Day10、Day11、Day12、Day13、Day14、Day19、Day23、Day28、中止時
（ただし、登録時/Day1 以外の観察時期は、入院/外来の状況を考慮して可能な限り実施する）

(10) PCR 検査

観察項目：唾液検体における SARS-CoV-2 ウイルス量
観察時期：登録時/Day1～Day28 まで連日。ただし、3.4.1 主要評価項目に定義するウイルス陰性化確認以降は実施不要とする。

(11) 血液学的検査

観察項目：白血球数、白血球分画（好中球数、好酸球数、好塩基球数、リンパ球数、単球数）、赤血球数、ヘモグロビン量、ヘマトクリット値、血小板数
観察時期：同意取得、スクリーニング時、Day4、Day8、Day11、Day14、Day19、中止時
（ただし、同意取得、スクリーニング時、以外の観察時期は、入院/外来の状況を考慮して可能な限り実施する）

(12) 血液生化学検査

観察項目：CRP、総蛋白、アルブミン、ALT、AST、γ-GTP、ALP、LDH、総ビリルビン、CK、電解質（Na、K、Cl）、BUN、CRE、血糖、プロカルシトニン
観察時期：同意取得、スクリーニング時、Day4、Day8、Day11、Day14、Day19、中止時
（ただし、同意取得、スクリーニング時、以外の観察時期は、入院/外来の状況を考慮して可能な限り実施する）

(13) 血液凝固検査

観察項目：フィブリノーゲン、PT、APTT、PT-INR、D-ダイマー
観察時期：同意取得、スクリーニング時、Day4、Day8、Day11、Day14、Day19、中止時
（ただし、同意取得、スクリーニング時、以外の観察時期は、入院/外来の状況を考慮して可能な限り実施する）

(14) 胸部単純 X 線検査又は胸部 CT 検査

観察項目：胸部単純 X 線検査又は胸部 CT 検査
観察時期：同意取得、スクリーニング時（Day8、Day14、中止時に診療上の必要性があり実施された場合には結果を収集する）

(15) 併用薬剤、併用療法

観察項目：併用薬剤、併用療法（6.4 項 参照）
観察方法：受診日の聴取・診察によって、上記確認する。
観察時期：同意取得日から試験終了又は中止日まで

(16) 有害事象
　観察項目：有害事象
　観察方法：受診日の聴取・診察及び患者からの報告によって、上記確認する。
　観察時期：治験薬初回投与日もしくは登録時/Day1 から試験終了又は中止日まで (7.2.1 有害事象の記録参照)

(17) 肺炎発症
　観察項目：肺炎発症の有無
　観察方法：治験責任医師又は治験分担医師が診察の結果肺炎を疑い、胸部単純 X 線検査又は胸部 CT 検査を実施し肺炎像を認めた場合、肺炎有りと判断する。
　観察時期：同意取得、スクリーニング時から試験終了又は中止日まで

(18) 生存状況
　観察項目：生存状況
　観察方法：来院、電話等で生存状況を確認する。
　観察時期：同意取得、スクリーニング時から Day28 相当日まで

6.3 試料の採取及び測定

(1) 試料の採取
　試料の採取は来院時に実施医療機関にて、もしくは被験者療養場所（自宅など）で行う。

(2) 中央測定を行う。
　以下の測定項目については、中央測定を行う。
　・唾液中 SARS-CoV-2 ウイルス量および陽性・陰性判定
　なお、測定および陽性・陰性判定は割り付け群について盲検下で中央臨床検査施設が行う。陰性判定は Ct > 40 とする。

6.3.1 唾液検体の取扱い

(1) 処理・保管方法
　中央測定用の唾液検体採取は、指定の採取容器を用いて行う。検体の処理・保管方法は、採取容器とともに提供される説明資料に従って行う。
(2) ラベル
　被験者が識別できる記載（氏名、イニシャルを除く）及び採取ポイントの情報を含むバーコードシールを明記する。
(3) 試料の輸送方法
　試料は、臨床検査施設へ輸送する。
(4) 結果報告
　各実施医療機関は、臨床検査施設より測定結果を入手する。

6.4 前治療及び併用薬・併用療法等

治療薬及び併用療法は、同意取得前 2 週から情報を収集する。但し、酸素療法に関わる情報は併用療法ではなく酸素投与の項目で収集する。

6.4.1 下痢の対処法

被験薬に特化した対処法は存在しないので、ロペラミドやタンニン酸製剤等の投与を行う。ただし、COVID-19 の症状としても下痢が認められることがあるため、慎重に判断すること。

6.4.2 併用が禁止される薬剤

本治験に影響を及ぼすと考えられる以下の薬剤についてランダム化時点から観察期間終了まで併用を禁止する。なお、やむを得ず他の薬剤を使用又は他の療法を施行した場合は、すべてその内容「薬剤名・治療法・期間（開始日、継続・終了日）、併用理由等」を症例報告書に記録する。

<<両群共通>>

他の臨床試験薬の投与、臨床試験機器の使用

新型コロナワクチン

<<被験薬投与群のみ>>

(1) CYP3A4 阻害により重篤な副作用が発現する恐れがある薬剤

トリアゾラム、ミダゾラム、アルプラゾラム、ピモジド、バッカク誘導体、アミオダロン塩酸塩、キニジン硫酸塩水和物、エレトリプタン臭化水素酸塩、エプレレノン、ブロナンセリン、スボレキサント、イバブラジン、チカグレロル、リバーロキサバン、アゼルニジピン

(2) CYP3A4 誘導作用を有し被験薬の薬剤血中濃度に影響を及ぼす薬剤

リファンピシン

(3) 被験薬との併用により被験薬及び／又は併用薬に影響を及ぼす薬剤

インジナビル硫酸塩エタノール付加物、サキナビル酸塩、リトナビル、ホスアンプレナビルカルシウム水和物、デラビルジン酸塩、リファブチン、エチニルエストラジオール又はノルエチステロンを含む経口避妊薬、フェノバルビタール、フェニトイン、カルバマゼピン、シンバスタチン、アトルバスタチン水和物、タクロリムス、シクロスポリン、エベロリムス、セイヨウオトギリソウ（St. John’s Wort、セント・ジョーンズ・ワート）含有食品、アジスロマイシン水和物、ボリコナゾール、オメプラゾール、CYP3A4 の基質となる薬剤（シルデナフィルクエン酸塩、タダラフィル、フルチカゾンプロピオン酸エステル、トラゾドン塩酸塩、ルラシドン、クエチアピン、コルヒチン、シロリムス、バルデナフィル、アプレピタント、ホスアプレピタント、イブルチニブ、エバスチン、エリグルスタット、テムシロリムス、ダサチニブ、ダルナビル、トルバプタン、ニソルジピン、フェロジピン、ブデソニド、マラビロク、リルピビリン、ロミタピド等）、サルメテロール、ネビラピン、ボセンタン、メサドン、ロスバスタチン、ワルファリン

6.5 過量投与

治験薬を過量投与した際は一般的な対症療法を行うこと。なお、被験薬は蛋白結合率が高いため、血液からの除去法として透析は不適切である。

6.6 被験者の管理

治験責任医師又は治験分担医師は被験者を連絡が取れる状態に置くこと。

被験者が来院しない場合、被験者の健康状態を確かめるため、治験責任医師又は治験分担医師はあらゆる手段を用いて被験者と連絡を取ることに努める。

6.7 治療方法遵守の確認

モニターは、カルテ等の原資料を確認、もしくはオフサイトモニタリングを実施することにより、適切に治験が実施されていることを確認する。

治験薬投与状況については、服薬日誌（付録 1）及び残薬等を必要に応じて確認する。

7 有害事象

7.1 有害事象発生時の対応

各実施医療機関の治験責任医師等は、治験薬初回投与日（被験薬投与群）／登録時/Day1（対症療法群）以降、被験者に有害事象を認めたときは、直ちに適切な処置を行うとともに、診療録ならびに症例報告書に記録する。

7.1.1 有害事象の定義

有害事象とは、患者に生じた好ましくないあらゆる医学的出来事のことであり、治験薬との因果関係の有無は問わない。原疾患の悪化（臨床検査値異常を含む）は有害事象として取り上げない。ただし、治験薬との因果関係が否定できない場合又は死亡に至った場合は、有害事象として取り扱う。

また、治験開始前より存在するその他の基礎疾患（合併症含む）による症状や臨床検査値、検査所見の異常は有害事象として取り上げない。原疾患に起因しない新たな症状や基礎疾患の悪化による症状や臨床検査値、検査所見の異常を認めた場合には、有害事象として取り上げる。

妊娠又は授乳の報告については「7.2.3 妊娠に関する報告」を参照する。

7.1.2 有害事象の報告

(1) 有害事象名は、原則として診断名・疾患名で症例報告書に記録する。診断名・疾患名が特定できない場合や、治験責任医師又は治験分担医師により診断名・疾患名としないことが妥当と判断された場合には、臨床症状又は徴候（臨床検査値の異常を含む）の名称を有害事象名として症例報告書に記録する。

(2) 臨床検査値異常が認められた場合、治験責任医師又は治験分担医師は当該異常を有害事象とするか判断する。有害事象としなかった場合は、その判断理由を記録する。

(3) 複数の臨床症状又は徴候（臨床検査値の異常を含む）がある有害事象（疾患）の随伴症状である場合、原則としてそれらを一つの有害事象として報告する。

7.1.3 重篤な有害事象

有害事象のうち、下記のいずれかの基準に該当する場合、重篤な有害事象と定義する。

1) 死亡
2) 障害
3) 死亡又は障害につながるおそれのある症例
4) 治療のため病院又は診療所への入院又は入院期間の延長が必要とされる症例
5) 1)～4)に準じて重篤である症例
6) 後世代における先天性の疾病又は異常

なお、同意取得前より予定していた療法又は検査を実施することのみを目的とした入院（例：手術や検査等）、また有害事象に伴う治療又は検査の目的以外の入院（例：健康診断等）は重篤な有害事象として取り扱わない。原疾患の悪化と判断される事象は有害事象として取り扱わない（「7.1.1 有害事象の定義」参照）が、上記 1) 死亡に該当する場合は、重篤な有害事象として報告する（「7.2.2 重篤な有害事象の報告」参照）。

7.1.4 有害事象の治験薬との因果関係

治験責任医師又は治験分担医師は被験者に関する知見、事象を取り巻く環境、考え得る他の全ての要因を考慮し、有害事象と治験薬との因果関係の有無を「あり」又は「なし」で評価する。評価にあたっては以下の基準を考慮する。

(1) 当該事象の発現時期と治験薬投与開始時期との時間的関連性
(2) 当該事象の経過、治験薬の投与中止又は投与再開（該当する場合）の影響
(3) 当該事象と治験薬又は類薬との既知の関連性
(4) 当該事象と本治験の対象疾患との既知の関連性
(5) 当該事象の発現率を増加させる既知の危険因子の存在又は併用薬の使用の有無
(6) 当該事象の発現との関連が確認されている、治験薬と無関係な因子の存在

7.1.5 有害事象の転帰

(1) 回復：症状、所見が消失又は有害事象発現以前の状態に戻った場合
(2) 軽快：症状、所見が消失又は有害事象発現以前の状態に回復する傾向が見られる場合
(3) 後遺症ありの回復：病気又はけがが原因の病態を残した状態で症状、所見がほぼ消失又は有害事象発現以前の状態にほぼ戻った場合
(4) 未回復：症状、所見が軽快せず不変の場合
(5) 死亡：当該有害事象により被験者が死亡した場合
(6) 不明：有害事象発現後、一度も転帰が確認できなかった場合

7.1.6 副作用

有害事象のうち、治験薬との因果関係の有無が「あり」と判断されたものを副作用とする。

7.1.7 有害事象の重症度の判定

有害事象の重症度は以下の基準に従って判定する。

軽度：日常生活に支障をきたさないと考えられる程度の有害事象（例：経過観察が可能)」<br>中等度：日常生活に支障をきたすと考えられる程度の有害事象（例：何らかの介入や治療が必要）<br>高度：日常生活を困難にすると考えられる程度の有害事象（例：高度かつ集中的な治療や全身介助が必要）

7.1.8 有害事象の重篤性の判定

有害事象の重篤度は、以下に分類する。

(1) 重篤である
(2) 重篤でない

7.1.9 有害事象の治療のためにとられた処置

有害事象を治療するために被験者に対して取られた処置は以下に分類される。

(1) 処置あり

(2) 処置なし

有害事象発現時に治験薬に対して取られた処置は以下に分類される。

(1) なし

(2) 休薬

(3) 中止

7.2 有害事象の記録と報告

7.2.1 有害事象の記録

治験責任医師又は治験分担医師は被験者の有害事象を評価し、以下の期間中に発現又は悪化した有害事象について、その内容、程度（重症度、重篤性)、発現日、消失日、処置、転帰、治験薬との因果関係等の必要事項を症例報告書に記録する。

・被験薬投与群：治験薬初回投与日から、治験薬最終投与日の 14 日後までに発現又は悪化した有害事象を収集する。また、治験薬最終投与日の 14 日後まで転帰調査を行い、有害事象終了日及び転帰に関して症例報告書に記録する。
・対症療法群：登録時/Day1 から、Day28 来院日もしくは中止時までに発現または悪化した有害事象を収集する。また、Day28 来院日もしくは中止時まで転帰調査を行い、有害事象終了日及び転帰に関して症例報告書に記載する。

なお、被験者の上記の有害事象収集期間の最後の日の時点で未回復の事象については、その時点まで、または「7.3 有害事象発現時の被験者のフォローアップ」に従って得られた経過を症例報告書に記録する。有害事象は、規制要件に準拠して規制当局へ報告する。これらの報告書の書式は規制要件に従う。

7.2.2 重篤な有害事象の報告

重篤な有害事象が発生した場合、治験責任医師は速やかに当該被験者について適切な処置の指示を行う。治験責任医師は、本治験期間中に被験者の重篤な有害事象、治験終了（中止）後に本治験との関連性が疑われる重篤な有害事象を知った場合は、最初に知り得た時点から24時間（1労働日）以内に「統一書式（医）12　重篤な有害事象に関する報告書」を作成し、所属する実施医療機関の長及び治験調整医師に提出する。また、治験調整医師は各実施医療機関の治験責任医師等と、当該重篤な有害事象の発生に関わる情報を共有する。

なお、上記の期限内に「重篤な有害事象に関する報告書」等の作成ができない場合には、口頭、電話等により重篤な有害事象について連絡を行い、「重篤な有害事象に関する報告書」等が作成でき次第、所属する実施医療機関の長及び治験調整医師に提出する。

その後、詳細報告を「統一書式（医）詳細記載用書式（書式12、14、19共通添付書式）」等の文書で提出する。この場合に、治験調整医師、実施医療機関の長又は治験審査委員会等から必要な情報を求められたときは、治験責任医師はこれに応じなければならない。

ただし、対症療法群に重篤な有害事象が発生した場合は、報告方法については治験調整医師、治験責任医師等と協議する。

7.2.3 妊娠に関する報告

被験薬投与群については、被験薬初回投与日から治験参加の期間までに女性被験者の妊娠が推定された場合、あるいは授乳が行われた場合には、これらすべての妊娠又は授乳について報告しなければならない。

対症療法群については、報告不要とする。

これらの情報は治験責任医師又は治験分担医師が転帰を知り得た場合、速やかに報告する。また、治験責任医師又は治験分担医師は当該被験者の妊娠に関する追跡調査を実施し、その転帰を報告する。妊娠の転帰が好ましくなく、治験薬との因果関係が疑われる場合には、治験薬投与からの経過時間の長さにかかわらず報告する。子の追跡も行い、当該治験薬との関連が疑われる情報を入手した場合は治験薬投与からの経過時間の長さにかかわらず報告する。その際、安全性情報の取扱いに関する標準業務手順書における「妊娠に関する報告書」（様式4）を用いて報告する。

妊娠の転帰が、先天異常、死産、人工流産又は自然流産の場合は、重篤な有害事象とみなし、重篤な有害事象と同様の報告期限及び方法で報告する（「7.2.2 重篤な有害事象の報告」参照）。

7.2.4 緊急報告

治験調整医師は規制要件に従い緊急報告すべき有害事象を治験責任医師又は治験分担医師及び実施医療機関の長ならびに規制当局へ報告する。そのため、治験責任医師又は治験分担医師は「7.2.2 重篤な有害事象の報告」で規定された手順に従い報告する。

7.3 有害事象発現時の被験者のフォローアップ

治験責任医師又は治験分担医師は、治験薬投与開始後、有害事象が認められた場合は最善の処置・治療を行う。有害事象等の発現のため、本治験の継続が困難と判断した場合は、治験を中止しその後の経過を観察する。なお、治験終了・中止時に治験薬との因果関係が否定できない有害事象が未回復の場合は、回復又は軽快するまで可能な限り観察を継続する。ただし、治験責任（分担）医師が本治験の影響は消失しており、被験者の安全性は十分確保され、それ以上の追跡調査は必要ないと判断した場合はこの限りではない。

7.4 効果安全性評価委員会

効果安全性評価委員会は、治験調整医師及び治験責任医師又は治験分担医師とは独立して設置され、「効果安全性評価委員会に関する標準業務手順書」に従い治験の全体的な安全性及び有効性を確認する。

7.5 医学的に重要な事象

7.5.1　薬物誘発性肝障害の可能性(Hy's Law)

薬物に曝露されて肝障害（トランスアミナーゼ値の上昇で判定される）の徴候を認めない人を「Tolerator」、一時的に肝障害を認めるが適応する人を「adaptor」と呼ぶ。一部の患者におけるトランスアミナーゼ値の上昇は、より重篤な潜在的転帰の前兆である。これらの患者は適応できないため、薬物

誘発性肝障害（DILI）と一般に称される進行性で重篤な肝障害を起こしやすい（「susceptible」と呼ぶ）。施設基準値上限（ULN）の 3 倍を超えるトランスアミナーゼ値の上昇を示す患者を頻繁にモニタリングし、「adaptor」なのか「susceptible」なのかを判断すること。

DILI 症例の多くでは、数日から数週間のうちに、AST 値や ALT 値の上昇が総ビリルビン(T-Bil)値の上昇（ULN の 2 倍超）に先立って認められる。一般に AST 値や ALT 値が ULN の 3 倍を超えて上昇すると、T-Bil 値が上昇する（すなわち、AST 値や ALT 値および T-Bil 値が同じ臨床検体において上昇する）。まれに、T-Bil 値の上昇が検出されるまでに、AST 値や ALT 値が低下していることがある。その場合においても DILI が疑われる。したがって、T-Bil 値に加えて AST または ALT のどちらかの値が異常に上昇し、以下に示す基準に合致する場合は、DILI が疑われる（Hy's Law に基づいて評価される）と考え、他に考えられる肝障害の原因が全て除外されていなくても、常に医学的に重要な事象とみなすこと。

DILI が疑われる症例の臨床検査値異常の閾値は、個々の患者のベースライン値および基礎疾患によって異なる。以下の臨床検査値異常を呈する患者は DILI が疑われる（Hy's Law）症例として、その原因を特定するためにさらなる評価を実施しなければならない：

・AST や ALT および T-Bil のベースライン値が基準値範囲内であった患者について、その後、AST 値または ALT 値が ULN の 3 倍超であることに加えて T—Bil 値が ULN の 2 倍超で溶血を認めない、かつアルカリフォスファターゼ値が ULN の 2 倍未満または値が不明である。
・AST または ALT または T-Bil のベースライン値が ULN を超えている患者については、ベースライン時点でどの値が ULN を超えているかにより、必要に応じて上述した定義に以下の閾値を使用する：
  ・AST または ALT のベースライン値がもともと基準値範囲を超えている場合：AST 値または ALT 値がベースライン値の 2 倍超かつ ULN の 3 倍超、または ULN の 8 倍超（いずれ小さい方）
  ・T-Bil のベースライン値がもともと基準値範囲を超えている場合：T-Bil 値がベースライン値から少なくとも ULN 分の増加、または ULN の 3 倍超（いずれか小さい方）

2~3 週間にわたり AST 値や ALT 値および T-Bil 値が別々に上昇した場合は、臨床判断に基づいて個別に評価すること。それでも Hy's Law の適用が疑われる症例かどうか断定できない症例については、治験調整事務局と協力して検討すること。

可能な限り速やかに、できれば、異常値に気づいてから 48 時間以内に、患者を治験実施医療機関に来院させ、評価を行うこと。この評価には、臨床検査、詳細な病歴および身体所見の評価を含めること。肝新生物（原発性又は二次性）の可能性を考慮すること。再検査において上述した AST 値や ALT 値および T-Bil 値の上昇に関する臨床検査基準に合致することが判明した全ての症例は、肝機能検査異常について、他の原因が明らかにならない限り、DILI が疑われる（Hy's Law）症例とみなす。肝機能検査異常の原因を解明するための検査結果がすべて揃っていなくても、このような DILI が疑われる（Hy's Law）症例は、医学的に重要な事象としてファイザーの安全性部門に報告する。

7.5.2 授乳期間中の治験薬曝露

授乳期間中の治験薬曝露について、治験調整医師は知り得てから 24 時間以内に所定の書式でファイザーの安全性部門に報告しなければならない。

7.5.3 職業的治験薬曝露

職業的治験薬曝露は、業務遂行中に被曝露者（医療従事者に限らない）が予期せず治験薬に接触することで発生し、その結果有害事象が発現する場合と発現しない場合がある。職業的治験薬曝露については、関連する有害事象発現の有無に関わらず、治験調整医師は知り得てから 24 時間以内にファイザーの安全性部門に報告する。この情報は本治験に登録される患者には関連しないため、症例報告書には記録しない。しかし、作成した書式の写しを治験実施医療機関の治験ファイルに保管する。

7.5.4 投薬過誤

投薬過誤は、誤った患者に対して、または誤った用量の治験薬が投与又は摂取されることによって生じる可能性がある。

投薬過誤には以下が含まれる：

・患者の治験薬への曝露に関わる投薬過誤

・潜在的な投薬過誤、または本治験に参加した患者であるか否かに関係なく本治験実施計画書で規定した方法以外の使用
・規定用量を超える用量で投与した場合

患者にこのような投薬過誤が生じた場合は、治験担当医師は治験調整医師に報告する。重篤な有害事象を伴う場合に限り、治験調整医師は24時間以内にファイザーの安全性部門に報告する。

8 統計解析

8.1 全般的事項

統計解析計画の詳細は、統計解析計画書に記載する。統計解析計画書において本治験実施計画書の概要を修正することがあるが、主要評価項目の定義や解析方法が変更される場合には、本治験実施計画書を改訂する。

データが固定された後に解析を行う。

全ての有効性評価において、Intention-To-Treat 解析対象集団における解析を主解析とし、参考として最大の解析対象集団（FAS）、治験実施計画書に適合した解析対象集団（PPS）における解析を行う。安全性の解析は、安全性解析対象集団（SAS）における解析を実施する。

統計解析の詳細は1症例目の割り付け実施に先立ち別途作成する統計解析計画書に記載する。

8.2 解析対象集団の定義

解析対象集団とその定義を以下に示す。解析対象集団はCONSORT 2010 Flow Diagramに従い、フローチャートとして表示する。

解析対象集団	定義
登録例	本治験に登録されたすべての被験者
割付例	スクリーニング例のうち、本治験に割付けられた被験者
安全性解析対象集団<br>Safety analysis set（SAS）	割り付け例とする。ただし、被験薬投与群に割り付けられたが1回も被験薬を投与されなかった被験者は、対症療法群として扱う。また、対症療法群として割り付けられたが、1回でも被験薬を投与された被験者は、被験薬投与群として取り扱う。
Intention-To-Treat 解析対象集団<br>（ITT 解析対象集団）	割り付け例とする。ただし、被験薬投与群に割り付けられた被験者は、1回も被験薬を投与されなかった場合も被験薬投与群として扱う。また、対症療法群として割り付けられた被験者は、被験薬を投与された場合も、対症療法群として取り扱う。
最大の解析対象集団<br>Full analysis set（FAS）	割り付け例とする。ただし、被験薬投与群に割り付けられたが1回も被験薬を投与されなかった被験者は、対症療法群として扱う。また、対症療法群として割り付けられたが、1回でも被験薬を投与された被験者は、被験薬投与群として取り扱う。
治験実施計画書に適合した解析対象集団<br>Per protocol set（PPS）	FASの内、データ固定前に決定された被験者の取扱い基準を満たす被験者。割付けられた投与群とは異なる投与群の用法・用量が用いられた被験者は、実際に投与した群として取り扱う

8.3 症例の取り扱い

治験調整医師は、1症例目の割り付け実施に先立ち、各症例の解析対象集団への採否を定めた被験者取り扱い基準、データの取扱い及び統計解析計画を確定する。

統計解析計画書の変更が必要な場合、統計解析計画書を改訂し、記録を残す。

8.4 被験者特性の解析

SAS、ITT解析対象集団、FAS、PPSそれぞれについて要約統計量を算出する。

8.5 有効性の解析

8.5.1 主要評価項目

主解析：割り付け群ごとのウイルス陽性者の割合について、両群のウイルス陰性化までの日数が等しいという帰無仮説の検定は、割付調整因子（重症度[無症状 vs.軽症]、年齢[60 歳未満 vs.60 歳以上]）を層とした層別ログランク検定により行う。ただし、各層の被験者数・陰性化イベント数が少ない場合など、2 つの因子を用いて適切に層別ログランク検定が行えないことが想定される場合には、群間比較を伴う検証的解析を行う前に群間比較に関わる情報がない下で作成する統計解析計画書で割付調整因子の扱いを定める。累積ウイルス陰性化曲線、ウイルス陰性化期間中央値、週次ウイルス陰性化割合などの推定は Kaplan-Meier 法を用いて行い、Brookmeyer and Crowley の方法を用いてウイルス陰性化期間中央値の信頼区間を求め、Greenwood の公式を用いてウイルス陰性化割合の信頼区間を求める。

副次解析：割り付け群のハザード比とその 95%信頼区間を、割付調整因子（重症度[無症状 vs.軽症]、年齢[60 歳未満 vs.60 歳以上]）を共変量とした Cox 比例ハザードモデルを用いて求め、さらに以下の帰無仮説について p 値を求める（スコア検定）：割付群間のハザード比 ＝1。

8.5.2 副次評価項目

time-to-event 型の評価項目：

割り付け群ごとのイベント発生までの日数が等しいという帰無仮説の検定を、割付調整因子（重症度[無症状 vs.軽症]、年齢[60 歳未満 vs.60 歳以上]）を層とした層別ログランク検定により行う。ただし、各層の被験者数・イベント数が少ない場合など、2 つの因子を用いて適切に層別ログランク検定が行えないことが想定される場合には、群間比較を伴う検証的解析を行う前に群間比較に関わる情報がない下で作成する統計解析計画書で割付調整因子の扱いを定める。累積イベント曲線、イベント発生時点の中央値、週次イベント発生割合などの推定は Kaplan-Meier 法を用いて行い、Brookmeyer and Crowley の方法を用いてイベント発生時点の中央値の信頼区間を求め、Greenwood の公式を用いてイベント発生割合の信頼区間を求める。

副次解析：割り付け群のハザード比とその 95%信頼区間を、割付調整因子（重症度[無症状 vs.軽症]、年齢[60 歳未満 vs.60 歳以上]）を共変量とした Cox 比例ハザードモデルを用いて求め、さらに以下の帰無仮説について p 値を求める（スコア検定）：割付群間のハザード比 ＝1。

割合の評価項目：

主解析：割り付け群のオッズ比とその 95%信頼区間を割り付け因子を共変量としたロジスティック回帰モデルを用いて求め、さらに以下の帰無仮説について p 値を求める（尤度比検定）：割付群間のオッズ比 ＝1。

8.6 安全性の解析

[解析対象集団]　SAS

(1) 有害事象について SOC/PT 別に発現例数、発現件数を集計する。別途、因果関係、重症度、重篤性により層別した発現例数、発現件数の集計を行う。
(2) 連続量の評価項目については、評価項目ごとに症例ごとの推移図を作成する。
(3) 評価項目ごとに、時点ごとの要約統計量を算出する。

9 原資料の特定/原資料等の直接閲覧

9.1 原資料の特定

原資料とは、元となる文書、データ及び記録（例：入院記録、診療録、検査ノート、メモ、服薬日誌及び評価用チェックリスト、心電図の記録データ、心エコー及びX線等の画像データ、及び薬剤部、検査部等の本治験に関与している医療技術部門で保管される記録等）をいう。

症例報告書に記録されるすべてのデータは、原資料と矛盾しないことが必要である。

但し本試験では、令和2年4月7日に厚生労働省医薬・生活衛生局医薬品審査管理課通知「治験における同意文書の保存に関する取扱いについて」により、被験者が触れる同意文書・投与日誌・症状日誌等の原資料の保管については、原資料となる元の文書の保管に代わり、原本との同一性を担保できるよう複写の手順を定めた上で作成した保証付き複写物（紙媒体/電磁的記録）を保管することも許容する。

9.2 外部測定機関から直接入手するデータ

臨床検査施設で測定された検査結果については電子データを手順書に従い直接入手する。原資料は実施医療機関にて保存される測定結果報告書とする。

9.3 画像評価の回収

本治験では診療録等の所見及び評価を収集する。画像データの回収は行わない。

9.4 原資料等の直接閲覧

治験調整医師が指名したモニターは GCP に従って直接閲覧を実施し、治験実施計画書の遵守状況及びデータの正確性を確認する。

症例報告書の内容と原資料を照合して矛盾がないかを確認する。なお、原資料となんらかの矛盾がある場合にはその理由を説明した記録を治験責任医師より入手する。実施医療機関におけるすべての記録は規制当局による査察及び治験審査委員会による調査の対象となる。なお、実施医療機関は、治験調整医師と協議の上、直接閲覧の方法、実施時期、原資料の特定及び閲覧項目を決定する。

10 治験の品質管理及び品質保証

10.1 治験の品質管理及び品質保証

治験調整医師は、治験調整医師が定める各標準業務手順書及び監査に関する標準業務手順書に従い、治験の品質管理及び品質保証を行う。

10.2 本治験における品質管理及び品質保証

治験調整医師は、治験実施計画書、各標準業務手順書及び本治験のモニタリング／監査計画書に従い、治験の品質を管理する。主な業務は以下のとおりである。

(1) 治験実施計画書の正確な理解、判定・評価の標準化を目的とし、治験責任医師、治験分担医師又は治験協力者に対し、治験開始時又は必要時に治験実施計画書説明会等を開催し説明を行う。
(2) 選択基準違反、除外基準違反を排除するために、外部の登録センターにより、症例登録票において被験者の適格性を確認する。
(3) モニターは、定期的に実施医療機関を訪問し、治験の適切な遂行について、本治験実施計画書及び GCP に従っていることを確認するとともにデータの正確性を確認する。
(4) 治験運営、データ収集、データ管理、統計解析、有害事象等の解析に関して記録及び報告を標準業務手順書に従い実施し、点検確認を行う。
(5) 本治験では治験が適正に行われている事を確保するため、治験調整医師は、品質管理を行うことにより、各開発業務の品質保証を行う。治験のシステム、及び治験が、薬事法、GCP 省令、治験実施計画書、標準業務手順書及び実施医療機関が定めた手順書を遵守して実施されているかを評価するため、医薬品の開発実施に関わる部門から独立した監査担当者が別に定める「監査の実施に関する標準業務手順書」に従い、治験に係る業務及び文書を体系的に調査・確認する。
(6) すべての原資料は治験調整医師又は国内外の規制当局の求めに応じ、閲覧することが可能である。

10.3 治験調整医師の監査と規制当局の査察

治験責任医師は、本治験実施計画書、GCP 及び適用される規制上の要件の遵守を徹底する目的で、治験調整医師の監査及び規制当局の査察を許可する。治験責任医師は、監査担当者及び査察担当者に対して治験記録を直接閲覧することに同意する。これらの業務を行う者は、評価したすべての個人の身元及び個人的な医学的情報を開示しない。

治験責任医師は、可能な限り監査及び査察の実施に協力するよう努める。治験責任医師は治験審査委員会又は規制当局より査察の連絡を受けた場合、直ちに治験調整医師へ連絡し、この査察のあらゆる結果を速やかに治験調整医師へ連絡する。治験責任医師は監査、査察で指摘されたすべての問題に対して適切な対策を行い、是正処置を講じる。

10.4 利益相反について

本治験は、厚生労働科学研究費事業研究費の公的な資金を用いて行われる。

本治験で使用される治験薬は日本たばこ産業株式会社およびファイザー株式会社より提供を受ける。

11 倫理

11.1 被験者への説明と同意の取得

11.1.1 説明文書の作成

治験責任医師は、被験者の同意を得るに際し、治験調整医師から提供を受けた説明文書の参考例及びその他の資料、情報をもとに、説明文書を作成する。作成された説明文書はその使用に先立ち各実施医療機関の治験審査委員会の承認を得る。

11.1.2 説明文書に記載すべき内容

(1) 治験が研究を伴うこと
(2) 治験の目的
(3) 治験の方法（治験の試験的側面、被験者の選択基準、及び各処置に割付けられる確率を含む）
(4) 被験者の治験への参加予定期間
(5) 治験に参加する予定の被験者数
(6) 予期される臨床上の利益及び危険性又は不便（被験者にとって予期される利益がない場合には、被験者にその旨を知らせなければならない）
(7) 患者を被験者にする場合には、当該患者に対する他の治療方法の有無及びその治療方法に関して予測される重要な利益及び危険性
(8) 治験に関連する健康被害が発生した場合に被験者が受けることのできる補償及び治療
(9) 治験への参加は被験者の自由意思によるものであり、被験者は、被験者の治験への参加を随時拒否又は撤回することができること。また、拒否・撤回によって被験者が不利な扱いを受けたり、治験に参加しない場合に受けるべき利益を失ったりすることはないこと
(10) 治験への参加の継続について被験者の意思に影響を与える可能性のある情報が得られた場合には速やかに被験者に伝えられること
(11) 治験への参加を中止させる場合の条件又は理由
(12) モニター、監査担当者、治験審査委員会及び規制当局が原医療記録を閲覧できること。その際、被験者の秘密は保全されること。また、同意文書に被験者が記名押印又は署名することによって閲覧を認めたことになること
(13) 治験の結果が公表される場合であっても、被験者の秘密は保全されること
(14) 被験者が費用負担をする必要がある場合にはその内容
(15) 被験者に金銭等が支払われる場合にはその内容（支払額算定の取決め等）
(16) 治験責任医師又は治験分担医師の氏名、職名及び連絡先
(17) 被験者が治験及び被験者の権利に関してさらに情報の入手を希望する場合又は治験に関連する健康被害が生じた場合に照会すべき又は連絡をとるべき実施医療機関の相談窓口
(18) 被験者が守るべき事項
(19) 治験の適否等について調査審議を行う治験審査委員会の種類、各治験審査委員会において調査審議を行う事項、各治験審査委員会の設置者の名称及び所在地、当該設置者に係る閲覧可能な情報等

(20) 治験審査委員会の手順書等が確認することができ、治験審査委員会の手順書等を確認したい場合には申し出てほしいこと及び治験審査委員会の手順書等を実施医療機関等のホームページで公表している場合は当該ホームページのアドレスを、公表していない場合は一般の閲覧に供していること

11.1.3 同意取得の時期と方法

(1) 治験責任医師又は治験分担医師は、被験者が治験に参加する前に、治験審査委員会の承認を得た説明文書を用いて十分に説明し、治験への参加について自由意思による同意を同意文書を用いて本人から得る。これらの手順の前に、被験者を治験に組み入れることはできない。
(2) 治験責任医師又は治験分担医師は、同意を得る前に、被験者が質問をする機会と、治験に参加するか否かを判断するのに十分な時間を与えなければならない。その際、当該治験責任医師、治験分担医師又は補足説明者としての治験協力者は、全ての質問に対して被験者が満足するように答える。
(3) 同意文書には、説明を行った治験責任医師又は治験分担医師、被験者が記名捺印又は署名し、各自日付を記入するものとする。なお、治験協力者が補足的な説明を行った場合には、当該治験協力者も記名捺印又は署名し、日付を記入する。
(4) 治験責任医師又は治験分担医師は、被験者が治験に参加する前に、記名捺印又は署名と日付が記入された同意文書の写し及びその他の説明文書を被験者に渡す。
(5) 治験責任医師等は、被験者に他に主治医がいるか否かを確認し、被験者の同意のもとに、主治医に被験者の治験への参加について治験薬の投与開始前に知らせる。
(6) 治験への参加の継続について、被験者の意思に影響を与える可能性のある情報が得られた場合には、治験責任医師又は治験分担医師は、当該情報を速やかに被験者に伝え、被験者の治験への参加の継続について被験者の意思を確認する。この場合にあっては、当該情報が被験者に伝えられたことを文書に記録する。

11.1.4 説明文書及び同意文書の改訂

(1) 被験者の同意に関連し得る新たな重要な情報が得られた場合には、治験責任医師は、速やかに当該情報に基づき説明文書及び同意文書を改訂し、予め治験審査委員会の承認を得る。
(2) 治験責任医師又は治験分担医師は、(1) に従い説明文書が改訂された場合には、すでに治験に参加している被験者に対しても改訂の都度、改訂の内容を速やかに伝え、治験に継続して参加するか否かについて、被験者の意思を確認するとともに、改訂された説明文書及び同意文書を用いて改めて説明し、治験への参加の継続について被験者から自由意思による同意を同意文書により本人から得る。
(3) 治験責任医師等は、新たに記名押印又は署名と日付を記入した同意文書の写し及び説明文書を被験者に渡さなければならない。

11.2 治験審査委員会

本治験は、治験を行うことの適否について、倫理的、科学的及び医学的妥当性の観点から治験審査委員会にて審査を受ける。また、重篤な有害事象発生時、治験実施計画書ならびに説明文書及び同意文書を改訂した場合には、治験を継続して行うことの適否について審査を受ける。なお、少なくとも 1 年に 1 回の頻度で治験審査委員会は治験が適切に実施されているか否かを継続的に審査する。このため、治験責任医師は、治験の現状の概要を実施医療機関の長に文書をもって提出する。

11.3 被験者の人権保護

(1) 治験責任医師及び治験分担医師は、被験者の選定にあたって、人権保護の観点から、選択基準及び除外基準に基づき、被験者の健康状態、症状、年齢、同意能力、治験責任医師等との依存関係、他の治験への参加の有無等を考慮のうえ、治験に参加を求めることの適否について慎重に検討する。
(2) 被験者の登録及び症例報告書における被験者の特定は被験者識別コードもしくは症例番号を用い、治験の実施に係る原資料類の直接閲覧、ならびに治験結果の公表においては、被験者の秘密が保全されるよう十分配慮する。
(3) 本治験に係る関係者（外部関係者も含む）は、被験者の個人情報保護について、適用される法令、条例を遵守する。また、関係者は、被験者の個人情報及びプライバシー保護に最大限の努力

を払い、当該治験を行う上で、知り得た個人情報を正当な理由なく漏らさない。関係者がその職を退いた後も同様とする。

11.4 同意撤回時の対応

(1) 治験責任医師等は、被験者からの治験参加の同意撤回の申し出があった場合は、その旨を診療録に記載する。

(2) 同意撤回があった場合、撤回時までに得たデータは解析に使用する。

11.5 治験終了後の対応

本治験の終了と同時に治験薬の投与は中止する。その後は被験者にもっとも適切と考える医療を提供する。

11.6 被験者に対する開示

各実施医療機関の長は、被験者より被験者等に係る個人情報についての開示を求められた場合は速やかに対応する。

12 データ及び試料の取扱いと保存

12.1 症例報告書の作成、変更・修正

適切かつ権限のある者（治験責任医師、治験分担医師又は治験協力者）がスクリーニングごとに症例報告書を作成する。症例報告書に記録されるすべてのデータは、症例報告書に直接記録されたデータを原資料とする場合を除き、原資料と矛盾しないことが必要である。症例報告書のデータを修正する場合には変更履歴を確実に残し、修正日、修正者及び修正理由を記録し、かつ修正前のデータが判読できなければならない。本治験実施計画書で規定された必要なデータのみを症例報告書に記録する。症例報告書に治験責任医師が署名した後に、治験調整医師へ提出し、実施医療機関はその写しを保存する。

本治験はEDCを用いてデータ入力を行う。治験責任医師等は各被験者の観察・検査・評価データを、内容確認後速やかにEDCに入力する。

なお、以下の条件が満たされている場合には、治験協力者が EDC の入力に関与しても差し支えない。

① 治験協力者の作成する範囲が原資料からのデータ転記のみであること。

② 治験責任医師が当該治験協力者の氏名と業務内容を記載した「統一書式　医　2　治験分担医師・治験協力者 リスト」を実施医療機関の長にあらかじめ提出し、その指名を受けていること。

③ 治験協力者が転記する箇所が明確で、治験責任医師がその内容を点検し間違いがないことを確認した上で入力すること。

EDCに入力したデータと原資料に矛盾がある場合には、治験責任医師はその理由を説明する記録を作成するか又はEDCを訂正する。治験責任医師等がEDCデータを訂正する場合、症例報告書の記載の手引きに従う。

なお、被験者より同意撤回があった場合は、撤回時までに得たデータは解析に使用する。

12.2 試料・情報の保管管理

12.2.1 試料・情報の取扱い

治験責任医師等は、治験に係る試料及び情報等を取り扱う際には被験者の個人情報とは関係のない番号（被験者識別コードもしくは症例番号）を付けて管理する。また、被験者と被験者識別コードもしくは症例番号を照合することができるよう対応表を作成する。作成した対応表は各実施医療機関で厳重に保管し院外へ持ち出す事はしない。

12.2.2 保管期間・場所

長崎大学大学院医歯薬学総合研究科　医療科学専攻　呼吸器内科学に集められた電子データはハードディスクにて可能な限り保管する。

本治験で得られた唾液検体は、国立感染症研究所にて可能な限り保管する。

各実施医療機関における対応表は、研究終了後、各実施医療機関の規定に従い保管する。

12.2.3 保管期間終了後の廃棄方法

長崎大学大学院医歯薬学総合研究科　医療科学専攻　呼吸器内科学において、保管期間が終了した紙媒体はシュレッダーにて廃棄、電子データは削除する。

国立感染症研究所に保管している唾液検体については国立感染症研究所の規定に従い医療廃棄物として廃棄する。

また、各実施医療機関に保管している対応表は個人情報に十分注意し、各実施医療機関の規定に従って廃棄する。

12.3 試料・情報の二次利用

本治験に関わる治験調整医師等が本治験で得られた試料および情報を異なる研究目的で使用する可能性または他の研究機関に提供する可能性があるが、その場合は、新たに研究計画書を作成し、適切な審査委員会等で審査、承認を得た上で、研究実施機関の手順に従い研究を開始する。インフォームド・コンセントは、研究の内容に合わせて適切な方法で行う。

12.4 記録の保存

12.4.1 治験審査委員会

治験審査委員会の設置者は、標準業務手順書、委員名簿（各委員の資格を含む）、委員の職業及び所属のリスト、提出された文書、会議の記録、その概要及び書簡等の記録を次の①又は②のうちいずれか遅い日まで保存する。ただし、治験調整医師がこれよりも長期間の保存を必要とする場合には、保存期間及び保存方法について治験審査委員会の設置者と協議する。これらの記録は、規制当局の要請に応じて提示できるようにしておかなければならない。

① 当該被験薬に係る製造販売承認日（開発の中止又は臨床試験の試験成績に関する資料が申請書に添付されないことが決定した場合にはその通知を受けた日）

② 治験の中止又は終了後 3 年が経過した日

12.4.2 実施医療機関

実施医療機関の長は、GCP 上医療機関において保存すべき治験に係る文書又は記録を次の①又は②のうちいずれか遅い日まで保存する。ただし、治験調整医師がこれよりも長期間の保存を必要とする場合には、保存期間及び保存方法について実施医療機関の長と協議する。記録の保存に際しては、それぞれの保存責任者を定めて保存する。実施医療機関の長又は記録の保存責任者は、これらの記録がこの保存義務期間中に紛失又は廃棄されることがないように、また、求めに応じて提示できるような措置を講じるものとする。

① 当該被験薬に係る製造販売承認日（試験の中止又は臨床試験の試験成績に関する資料が申請書に添付されないことが決定した場合にはその通知を受けた日）

② 治験の中止又は終了後 3 年が経過した日

12.4.3 治験責任医師又は治験分担医師

治験責任医師又は治験分担医師は、治験に係る文書又は記録を実施医療機関の長の指示に従って保存する。

12.4.4 治験調整医師

治験調整医師は、治験調整事務局が作成する標準業務手順書に従い GCP 上治験調整医師が保存すべき治験に係る文書又は記録を次の①又は②のうちいずれか遅い日までの間保存する。ただし、治験が中止された場合には、治験中止届出日から 3 年が経過した日までとする。

① 当該被験薬に係る製造販売承認日から 5 年が経過した日。ただし、医薬品医療機器等法の規定により承認後の再審査を受けなければならない医薬品で、かつ再審査が終了するまでの期間が 5 年を超えるものについては、再審査が終了する日。

② 最後の治験終了届の提出日から 15 年が経過した日。

なお、治験調整医師は、実施医療機関の長又は治験審査委員会の設置者が保存すべき治験に係る文書又は記録について、その保存の必要がなくなった場合には、その旨を実施医療機関の長又は実施医療機関の長を経由して治験審査委員会の設置者に対して通知する。

13 金銭の支払い及び健康被害補償

13.1 治験の費用

本治験で使用する治験薬は、日本たばこ産業株式会社およびファイザー株式会社から無償提供される。

本治験は厚生労働科学研究費事業研究費より資金を得て実施する。

13.2 健康被害補償

本治験は、これまでの報告に基づいて科学的に計画され、慎重に行われるものである。もし、本治験期間中あるいは終了後に被験者に健康被害が生じた場合には、通常の診療と同様に保険診療内で医師が適切な診察と治療を行う。

本治験に起因した健康被害が生じた場合、本治験は保険に加入しているため、保険の範囲内での補償がなされる。ただし、健康被害が被験者自身の故意又は重大な過失により引き起こされたことが証明された場合は補償を減額する又は補償しないことがある。また、新たに発生した健康被害やもともと患っている疾病の悪化が本治験とは因果関係がない場合は補償の対象としない。

14 成果の帰属と公表に関する取り決め

治験調整医師は、本治験により得られた情報を本治験薬の医薬品製造販売承認申請等の目的で自由に使用することができる。

本治験により得られた情報を、治験責任医師等が専門の学会等、外部に公表する場合には事前に治験調整医師の文書による承諾を得るものとする。

15 治験実施計画書の変更

15.1 治験実施計画書の改訂

(1) 治験調整医師は、治験の進行中に治験実施計画書に変更の必要性が生じた場合は、治験責任医師、効果安全性評価委員会治験調整事務局と協議して変更内容を決定し、変更内容とその理由について文書により速やかに治験責任医師との合意を得る。治験審査委員会の意見に基づき実施医療機関の長の指示により修正する場合も同様とする。

(2) 治験調整医師は、改訂された治験実施計画書及び該当する場合は改訂された症例報告書を速やかに実施医療機関の長に提出する。実施医療機関の長の許可を受けるまで治験計画を変更しての治験実施及び、変更された内容の被験者への説明は行わない。

15.2 治験実施計画書からの逸脱・変更

(1) 治験責任医師又は治験分担医師は、治験責任医師が治験調整医師との事前の文書による合意及び治験審査委員会の事前の審査に基づく文書による承認を得ることなく、治験実施計画書からの逸脱又は変更を行わない。

(2) ただし、治験責任医師又は治験分担医師は、被験者の緊急の危機を回避する等、医療上やむをえない事情のために治験調整医師との事前の合意及び治験審査委員会の事前の承認なしに治験実施計画書からの逸脱又は変更を行うことができる。この際、治験責任医師は、治験実施計画書から逸脱した行為をすべて記録し、その理由等を説明した記録を治験調整医師及び当該実施医療機関の長に提出し、その写しを保存する。

(3) 治験責任医師は、前項における治験実施計画書からの逸脱、又は変更の内容及び理由が適切と判断する場合、「15.1 治験実施計画書の改訂」に従い治験実施計画書の改訂について治験調整医師と協議し、合意する。その案を可能な限り早急に実施医療機関の長に提出し、治験審査委員会の承認を得る。

(4) 治験責任医師は、治験の実施に重大な影響を与え、又は被験者の危険を増大させるような治験のあらゆる変更について、治験調整医師、実施医療機関の長及び治験審査委員会に速やかに報告書を提出する。

16 治験の中止・中断・終了

16.1 治験の中止・中断

治験調整医師は、下記の事項に該当する場合は、治験の一部及び全体の実施の継続の可否を検討する。なお、個々の被験者の中止基準については、「4.3 中止基準」に記載した。

16.1.1 治験全体の中止・中断

- ・治験薬・治験で実施する治療法の安全性等に関して重大な情報が得られたとき。
- ・被験者の組入れが困難で、予定被験者数に達することが極めて困難であると判断されたとき。
- ・治験薬・治験で実施する治療法の効果不十分により倫理的に継続することが困難であると判断されたとき。
- ・治験審査委員会により、治験実施計画書等の変更の指示があり、これを受け入れることが困難と判断されたとき。

上記の場合、治験調整医師は治験責任医師と協議し、治験の中止・中断及びその理由の詳細を実施医療機関の長及び規制当局に速やかに文書で通知する。実施医療機関の長は治験責任医師及び治験審査委員会にその旨を通知し、文書で詳細を説明する。治験責任医師又は治験分担医師は被験者にその旨を通知し、適切な治療及び事後処理を保証する。

16.1.2 各実施医療機関における治験の中止・中断

(1) 治験調整医師による中止

治験調整医師は、治験責任医師、治験分担医師又は実施医療機関が、GCP、本治験実施計画書又は治験の契約に重大又は継続して違反することにより適正な治験の実施に支障を及ぼした場合には、当該実施医療機関との契約を解除し、当該実施医療機関での治験を中止する。

(2) 治験責任医師による中止・中断

治験責任医師が治験を中止又は中断した場合、治験責任医師は当該実施医療機関の長へ速やかにその旨を通知するとともに、詳細を文書にて説明する。当該実施医療機関の長は治験調整医師及び治験審査委員会に対し、速やかにその旨を文書で通知するとともに、詳細を文書にて説明する。

(3) 治験審査委員会による中止・中断

実施中の治験の継続審査等において、治験審査委員会が、有害事象等何らかの医学的情報又は倫理的判断に基づいて既に承認した事項の取消の決定を下し、その旨が当該実施医療機関の長に通知された場合、当該実施医療機関の長は、これに基づく指示、決定を治験審査委員会の取消に関する日付入り文書の写しとともに、治験責任医師及び治験調整医師に文書で詳細に説明する。

16.2 治験の終了

治験が終了した場合には、治験責任医師は実施医療機関の長にその旨を文書で通知し、治験結果の概要を文書（治験終了報告書）で報告する。実施医療機関の長は、治験審査委員会及び治験調整医師に対し、速やかに終了の旨を文書で通知するとともに、治験終了報告書に基づき、治験結果の概要を報告する。

17 治験実施体制

本治験は別紙 1 に記載する体制で実施する。

18 参考資料及び文献

1) World Health Organization: Novel coronavirus (2019-nCoV) situation report-1. 21 January 2020. https://www.who.int/docs/default-source/coronaviruse/situation-reports/20200121-sitrep-1-2019-ncov.pdf?sfvrsn=20a99c10_4
2) Chan JF, et al: Middle East respiratory syndrome coronavirus: another zoonotic betacoronavirus causing SARS-like disease. Clin Microbiol Rev. 2015;28:465-522.
3) Zhu N, et al: A novel coronavirus from patients with pneumonia in China, 2019. N Engl J Med. 2020;382:727-733.

4) Guan W, et al: Clinical Characteristics of Coronavirus Disease 2019 in China.N Engl J Med. 2020;382:1708-1720
5) World Health Organization: Novel coronavirus (2019-nCoV) situation report-46. 6 May 2020. https://www.who.int/docs/default-source/coronaviruse/situation-reports/20200306-sitrep-46-covid-19.pdf?sfvrsn=96b04adf_4
6) Wang D, et al: Clinical characteristics of 138 hospitalized patients with 2019 novel coronavirus-infected pneumonia in Wuhan, China. JAMA. 2020;323:1061-1069
7) Cai Q, et al: Experimental Treatment with Favipiravir for COVID-19: An Open-Label Control Study. Engineering. 2020 Mar 18[Epub ahead of print]
8) Grein J, et al: Compassionate Use of Remdesivir for Patients with Severe Covid-19. N Engl J Med. 2020 Apr 10[Epub ahead of print]
9) Matsuyama S, et al: The inhaled corticosteroid ciclesonide blocks coronavirus RNA replication by targeting viral NSP15. bioRxiv 2020.
10) Contini A: Virtual screening of an FDA approved drugs database on two COVID-19 coronavirus proteins. ChemRxiv 2020.
11) Gautret P, et al: Hydroxychloroquine and azithromycin as a treatment of COVID-19: results of an open-label non-randomized clinical trial. Int J Antimicrob Agents. 2020 Mar 20[Epub ahead of print]
12) Ohashi H, et al: Multidrug treatment with nelfinavir and cepharanthine against COVID-19. BioRxiv. 2020.
13) Liu Y, et al: Viral dynamics in mild and severe cases of COVID-19. Lancet Infect Dis.2020 Mar 19[Epub ahead of print]
14) 岡崎医療センターにおける SARS-CoV-2 無症状病原体保有者の PCR 陰性化状況（2020.3.13） http://www.kansensho.or.jp/uploads/files/topics/2019ncov/covid19_casereport_200313.pdf
15) Wu Z, et al: Characteristics of and Important Lessons From the Coronavirus Disease 2019 (COVID-19) Outbreak in China. JAMA. 2020 Feb 24[Epub ahead of print]
16) B. E. Young et al., Epidemiologic Features and Clinical Course of Patients Infected With SARS-CoV-2 in Singapore. Jama, (2020).
17) L. Zou et al., SARS-CoV-2 Viral Load in Upper Respiratory Specimens of Infected Patients. N Engl J Med, (2020).
18) R. Wolfel et al., Virological assessment of hospitalized patients with COVID-2019. Nature, (2020).
19) F. X. Lescure et al., Clinical and virological data of the first cases of COVID-19 in Europe: a case series. Lancet Infect Dis, (2020).
20) J. Y. Kim et al., Viral Load Kinetics of SARS-CoV-2 Infection in First Two Patients in Korea. J Korean Med Sci 35, e86 (2020).
21) Kim KS, et al: Modelling SARS-CoV-2 Dynamics: Implications for Therapy. MedRxiv. 2020.
22) Royal College of Physicians National Early Warning Score (NEWS) 2: Standardising the assessment of acute illness severity in the NHS. Updated reporting of a work party London: RCP, 2017. https://www.rcplondon.ac.uk/projects/outputs/national-early-warning-score-news-2.
23) Laure SA, et al: The Incubation Period of Coronavirus Disease 2019 (COVID-19) From Publicly Reported Confirmed Cases: Estimation and Application. Ann Intern Med. 2020;172:577-582.

19. 治験実施体制

19.1 治験調整委員会

治験調整医師（代表）

長崎大学大学院医歯薬学総合研究科 臨床感染症学分野　宮崎　泰可
【連絡・問い合わせ先】
〒852-8501 長崎県長崎市坂本 1-7-1
Tel: 095-819-7273/ e-mail: taiga-m@nagasaki-u.ac.jp

千葉大学医学部附属病院 臨床試験部　花岡　英紀
【連絡・問い合わせ先】
〒260-8677　千葉市中央区亥鼻 1-8-1
Tel：043-226-2737/e-mail： hanaoka.hideki@faculty.chiba-u.jp

千葉大学医学部附属病院 臨床試験部　黒川　友哉
【連絡・問い合わせ先】
〒260-8677　千葉市中央区亥鼻 1-8-1
Tel：043-226-2737/e-mail： t-kurokawa@chiba-u.jp

長崎大学病院 臨床研究センター　山本　弘史
【連絡・問い合わせ先】
〒852-8501 長崎県長崎市坂本 1-7-1
Tel: 095-819-7726/ e-mail: hiroshiy@nagasaki-u.ac.jp

長崎大学病院 臨床研究センター　細萱　直希
【連絡・問い合わせ先】
〒852-8501 長崎県長崎市坂本 1-7-1
Tel: 095-819-7726/ e-mail: nhosogaya@nagasaki-u.ac.jp

19.2 治験調整事務局

長崎大学病院 臨床研究センター　治験調整事務局責任者　福重　友理
【連絡・問い合わせ先】
〒852-8501 長崎県長崎市坂本 1-7-1
Tel: 095-819-7256/ e-mail: yurifukushige@nagasaki-u.ac.jp

長崎大学病院 臨床研究センター　竹森　幸子
【連絡・問い合わせ先】
〒852-8501 長崎県長崎市坂本 1-7-1
Tel: 095-819-7726/ e-mail: takemori-s@nagasaki-u.ac.jp

千葉大学医学部附属病院　臨床試験部　藤原　忠美
【連絡・問い合わせ先】
〒260-8677　千葉市中央区亥鼻 1-8-1
Tel：043-202-5730/e-mail：t-fujiwara@chiba-u.jp

千葉大学医学部附属病院 臨床試験部 堀　真琴
【連絡・問い合わせ先】
〒260-8677　千葉市中央区亥鼻 1-8-1
Tel：043-226-2737/e-mail：makotohori@chiba-u.jp

19.3 実施医療機関および治験責任医師

所属・連絡先	職名・氏名
長崎大学病院 呼吸器内科<br>〒852-8501 長崎県長崎市坂本 1-7-1<br>Tel：095-819-7200（代表）	医師<br>宮崎　泰可
東京大学医科学研究所附属病院 感染免疫内科<br>〒108-8639 東京都港区白金台 4-6-1<br>Tel：03-3443-8111（代表）	医師<br>四柳　宏
国際医療福祉大学 成田病院 感染症科<br>〒286-8520 千葉県成田市畑ヶ田 852<br>Tel：0476-35-5600（代表）	医師<br>加藤　康幸
埼玉医科大学病院 感染症科・感染制御科<br>〒350-0495 埼玉県入間郡毛呂山町毛呂本郷 38<br>Tel：049-276-1111（代表）	医師<br>樽本　憲人
大阪市立十三市民病院 呼吸器内科<br>〒532-0034 大阪府大阪市淀川区野中北 2-12-27<br>Tel：06-6150-8000（代表）	医師<br>白石　訓
福岡大学筑紫病院　呼吸器内科<br>〒818-0067 福岡県筑紫野市俗明院 1-1-1<br>Tel：092-921-1011（代表）	医師<br>石井　寛
埼玉医科大学総合医療センター　感染症科・感染制御科<br>〒350-8550 埼玉県川越市鴨田 1981 番地<br>Tel：049-228-3400（代表）	医師<br>大野　秀明
産業医科大学病院　呼吸器内科<br>〒807-8556 福岡県北九州市八幡西区医生ケ丘 1-1<br>Tel：093-603-1611（代表）	医師<br>矢寺　和博

富山大学附属病院　感染症科<br>〒930-0194 富山県富山市杉谷 2630 番地<br>Tel：076-434-2281（代表）	医師<br>山本　善裕
大阪市立大学医学部附属病院　感染症内科<br>〒545-8586 大阪府大阪市阿倍野区旭町 1-5-7<br>Tel：06-6645-2121（代表）	医師<br>掛屋　弘

19.4 効果安全性評価委員会

(1) 効果安全性評価委員会の主な役割

別途治験調整医師が作成する手順書に従い下記の業務を行う。

1)　治験進行中の評価
2)　安全性評価項目の評価
3)　重要な有効性評価項目の評価
4)　重篤かつ予測できない有害事象の評価
5)　上記 1)～4)の結果より治験の継続、変更、中止又は中断等の提言

(2) 効果安全性評価委員会構成メンバーの氏名、職名、連絡先

所属・連絡先	職名・氏名
長崎大学病院 リウマチ・膠原病内科<br>〒852-8501 長崎県長崎市坂本 1-7-1<br>Tel：095-819-7200（代表）	医師<br>川上　純
長崎大学病院 薬剤部<br>〒852-8501 長崎県長崎市坂本 1-7-1<br>Tel：095-819-7200（代表）	薬剤師<br>兒玉　幸修
久留米大学バイオ統計センター<br>〒830-0011　福岡県久留米市旭町 67 医学部 B 棟 7 階<br>Tel： 0942-31-7835	生物統計家<br>室谷　健太

19.5 治験薬提供者

日本たばこ産業株式会社

【連絡・問い合わせ先】

〒105-8422　東京都港区虎ノ門 2-2-1

Tel：03-3582-3111（代表）

ファイザー株式会社

【連絡・問い合わせ先】

〒10017　米国ニューヨーク州ニューヨーク市 42 番通り 235E

Tel: +1-212-733-2323

19.6 開発業務受託機関

19.6.1　治験薬の保管・配送

(1)主な委託業務内容

1)　治験薬の保管、配送

(2)業務受託機関の名称、所在地

名称：三菱倉庫株式会社

所在地：〒103-8630 東京都中央区日本橋 1-19-1

19.6.2　モニタリングの実施

(1)主な業務内容

1)　モニタリング業務

(2)業務実施機関の名称、所在地及び責任者

名称：千葉大学医学部附属病院 臨床試験部モニタリング室

所在地：〒260-8677 千葉県千葉市中央区亥鼻 1-8-1

モニタリング責任者：藤居　靖久

19.6.3　データマネジメントの実施

(1) 主な業務内容

1)　データマネジメント業務

(2) 業務実施機関の名称、所在地及び責任者

名称：千葉大学医学部附属病院 臨床試験部データマネジメント室

所在地：〒260-8677 千葉県千葉市中央区亥鼻 1-8-1

データマネジメント責任者：花輪　道子

19.6.4　統計解析の実施

(1) 主な業務内容

1)　統計解析業務

(2) 業務実施機関の名称、所在地及び責任者

名称：千葉大学医学部附属病院 臨床試験部生物統計室

所在地：〒260-8677 千葉県千葉市中央区亥鼻 1-8-1

統計解析責任者：川崎　洋平

19.6.5　総括報告書の作成

(1) 主な委託業務内容

1)　総括報告書の作成業務

(2) 業務受託機関の名称、所在地

名称：大阪大学医学部附属病院 未来医療開発部 未来医療センター
所在地：〒565-0871 大阪府吹田市山田丘 2-2

19.6.6 監査の実施

(1) 主な委託業務内容
1) 監査業務
(2) 業務受託機関の名称、所在地
名称：大阪大学医学部附属病院 未来医療開発部 未来医療センター
所在地：〒565-0871 大阪府吹田市山田丘 2-2

19.6.7 臨床検査施設

(1) 主な委託業務内容
1) 唾液 PCR 検査業務
(2) 業務受託機関の名称、所在地
名称：国立感染症研究所
所在地：〒162-8640 東京都新宿区戸山 1-23-1

19.6.8 ウイルス動態パラメータ（ウイルス量の曲線下面積、ウイルス量の半減期）の算出

(1) 主な業務内容
ウイルス動態パラメータ（ウイルス量の曲線下面積、ウイルス量の半減期）の算出
(2) 業務実施機関の名称、所在地及び責任者
名称：九州大学理学研究院生物科学部門数理生物学教室
所在地：〒819-0395 福岡県福岡市西区元岡 744 番地
解析責任者:岩見　真吾
